# Rapid whole genome sequencing methods for RNA viruses

**DOI:** 10.3389/fmicb.2023.1137086

**Published:** 2023-02-23

**Authors:** Masayasu Misu, Tomoki Yoshikawa, Satoko Sugimoto, Yuki Takamatsu, Takeshi Kurosu, Yukiteru Ouji, Masahide Yoshikawa, Masayuki Shimojima, Hideki Ebihara, Masayuki Saijo

**Affiliations:** ^1^Department of Virology I, National Institute of Infectious Diseases, Tokyo, Japan; ^2^Department of Pathogen, Infection and Immunity, Nara Medical University, Nara, Japan

**Keywords:** RNA virus, next-generation sequencing – NGS, MinION nanopore device, rapid amplification of cDNA ends (RACE), rolling circle amplification (RCA), whole genome sequencing (WGS)

## Abstract

RNA viruses are the etiological agents of many infectious diseases. Since RNA viruses are error-prone during genome replication, rapid, accurate and economical whole RNA viral genome sequence determination is highly demanded. Next-generation sequencing (NGS) techniques perform whole viral genome sequencing due to their high-throughput sequencing capacity. However, the NGS techniques involve a significant burden for sample preparation. Since to generate complete viral genome coverage, genomic nucleic acid enrichment is required by reverse transcription PCR using virus-specific primers or by viral particle concentration. Furthermore, conventional NGS techniques cannot determine the 5′ and 3′ terminal sequences of the RNA viral genome. Therefore, the terminal sequences are determined one by one using rapid amplification of cDNA ends (RACE). However, since some RNA viruses have segmented genomes, the burden of the determination using RACE is proportional to the number of segments. To date, there is only one study attempting whole genome sequencing of multiple RNA viruses without using above mentioned methods, but the generated sequences’ accuracy compared to the reference sequences was up to 97% and did not reach 100% due to the low read depth. Hence, we established novel methods, named PCR-NGS and RCA-NGS, that were optimized for an NGS machine, MinION. These methods do not require nucleic acid amplification with virus-specific PCR primers, physical viral particle enrichment, and RACE. These methods enable whole RNA viral genome sequencing by combining the following techniques: (1) removal of unwanted DNA and RNA other than the RNA viral genome by nuclease treatment; (2) the terminal of viral genome sequence determination by barcoded linkers ligation; (3) amplification of the viral genomic cDNA using ligated linker sequences-specific PCR or an isothermal DNA amplification technique, such as rolling circle amplification (RCA). The established method was evaluated using isolated RNA viruses with single-stranded, double-stranded, positive-stranded, negative-stranded, non-segmented or multi-segmented genomes. As a result, all the viral genome sequences could be determined with 100% accuracy, and these mean read depths were greater than 2,500×, at least using either of the methods. This method should allow for easy and economical determination of accurate RNA viral genomes.

## 1. Introduction

Viral infectious diseases are a significant threat to public health and the stability of the world’s economic infrastructure. In particular, RNA viruses are primary etiological agents of emerging human pathogens, accounting for 44% of all emerging infectious diseases (Carrasco-Hernandez et al., 2017).

Rapid and accurate whole viral genome sequence determination is highly demanded in RNA viral infectious diseases research since RNA viruses that encode RNA-dependent RNA polymerase lacking proofreading are more prone to mutation than DNA viruses (Sanjuan et al., 2010; Sanjuan and Domingo-Calap, 2016). In fact, during the ongoing COVID-19 pandemic caused by an RNA virus, SARS-CoV-2, a large amount of sequencing data on SARS-CoV-2 variant genomes has been shared on a global scale database (Shu and McCauley, 2017; Hadfield et al., 2018). The data contribute to epidemiological studies, such as tracking epidemic strains and new variants and developing treatment and prevention methods.

Next-generation sequencing (NGS) techniques generally perform whole viral genome sequencing due to extraordinary high*-*throughput sequencing capacity. However, even if NGS techniques are used, determining the whole genome sequence of RNA viruses is cumbersome and time-consuming for sample preparation. Since to generate complete viral genome coverage, genomic nucleic acid enrichment is required by reverse transcription PCR (RT-PCR) using virus-specific primers or by viral particle concentration using ultracentrifugation or centrifugal ultrafiltration (Kapoor et al., 2008; Thurber et al., 2009). So far, there is only one study attempting whole genome sequencing of multiple RNA viruses without using above mentioned methods for enriching the viral genomic nucleic acid (Wongsurawat et al., 2019). The study used an NGS machine, MinION from Oxford Nanopore Technologies, which is portable and inexpensive yet provides accurate consensus sequencing (Jain et al., 2016). However, the results of mean read depth aligned to viral reference genomes varied between 10× and 700×, and the mapped reads to the viral genomes were 0.04–1.72% of the total reads. Presumably, this is why the generated sequences’ accuracy compared to the reference sequences was up to 97% and did not reach 100% (Wongsurawat et al., 2019).

Furthermore, conventional NGS techniques cannot determine the 5′ and 3′ terminal sequences of the RNA viral genome. However, the terminal sequences play essential roles in viral propagation, and their sequence information is vital. Therefore, the terminal sequences are determined one by one using a so-called method, Rapid amplification of cDNA ends (RACE). However, in addition to RACE itself being a labor-intensive method, some RNA viruses contain segmented genomes, so the burden of the determination using RACE increases in proportion to the number of segments.

Hence, this study has established novel methods to determine the whole genome sequence of various RNA viruses, including positive, negative, double-stranded, and segmented genomes, with high read depth. The methods were optimized for MinION and did not require specific PCR primers, physical viral particle enrichment, or even RACE. Furthermore, the method does not require special reagents and can be easily performed using only commercially available reagents. The consensus sequence of viruses utilized in this study could be determined with 100% accuracy through the implementation of the established methods. The established methods will advance the viral genome determination method in NGS and contribute to virology research.

## 2. Materials and methods

### 2.1. Cells and viruses

Vero, Vero E6, Vero.DogSLAMtag, which is stably expressing a CDV receptor, canine SLAM (Seki et al., 2003; Sakai et al., 2013), MDCK and 293T cells were grown in Dulbecco’s Modified Eagle’s Medium (DMEM; catalog number 041-30081, Wako, Osaka, Japan) supplemented with 5% heat-inactivated fetal bovine serum (FBS), 100 U/ml penicillin, and 100 μg/ml streptomycin (catalog number 15140122, Thermo Fisher Scientific, Waltham, MA). In some cases, an antibiotic for *Mycoplasma* spp., BIOMYC-3 (catalog number PK-CC03-038-1D, Takara Bio, Shiga, Japan), was added to the culture medium at 100-fold dilution. The working stocks of RNA viruses were prepared as described and were aliquoted and stored at −80°C.

Lymphocytic choriomeningitis virus (LCMV) strain WE (Genbank Accession Numbers LC413283 and LC413284) was propagated in Vero cells at a multiplicity of infection (MOI) of 0.01. The culture supernatants were harvested at 4 days post-infection. The infectious dose was determined using Vero cells with the standard 50% tissue culture infectious dose (TCID_50_) assay, with visualization of infection on the wells in a 96-well plate by an indirect immunofluorescence assay (IFA), as described previously (Taniguchi et al., 2020).

Severe fever with thrombocytopenia syndrome virus (SFTSV) strain YG-1 (Genbank Accession Numbers AB817979, AB817987, and AB817995) was propagated in Vero cells at an MOI of 0.01. The culture supernatants were harvested at full cytopathic effect (CPE). The infectious dose was determined with the standard TCID_50_ assay, with visualization of infection on the wells in a 96-well plate by an IFA, as described previously (Takahashi et al., 2014).

Influenza A virus (IAV) strain H1N1 A/PR/8/34 (Genbank Accession Numbers LC662537, LC662538, LC662539, LC662540, LC662541, LC662542, LC662543, and LC662544) purchased from ATCC was propagated in MDCK cells with the addition of 1.0 μg/ml trypsin (catalog number 207-19183, Fujifilm Wako Pure Chemical Corporation, Osaka, Japan) in DMEM and passaged twice at an MOI of 0.01. The culture supernatants were harvested 3 days post-infection, and the infectious dose was determined using MDCK cells with the standard TCID_50_ assay in a 96-well plate.

Canine distemper virus (CDV) strain CYN07-dV (Genbank Accession Number AB687720) was propagated in Vero.DogSLAMtag cells at an MOI of 0.01. The cells and culture supernatants were harvested at full CPE and frozen and thawed twice, which is necessary to release the cell-associated virus into the culture supernatant. The samples were centrifuged at 1,000 ×*g* for 10 min, and the infectious dose was determined with the standard TCID_50_ assay in a 96-well plate.

Severe acute respiratory syndrome coronavirus 2 (SARS-CoV-2) strain 2019-nCoV/Japan/TY/WK-521/2020 (GISAID ID: EPI_ISL_408667) was propagated in VeroE6 cells stably expressing transmembrane serine protease TMPRSS2 (VeroE6/TMPRSS2) (Matsuyama et al., 2020) at an MOI of 0.1. The culture supernatants were harvested at full CPE, and the infectious dose was determined using VeroE6/TMPRSS2 cells with the standard TCID_50_ assay in a 96-well plate.

Pteropine orthoreovirus (PRV) strain Miyazaki-Bali/2007 (Genbank Accession Numbers AB908278.1, AB908279.1, AB908280.1, AB908281.1, AB908282.1, AB908283.1, AB908284.1, AB908285.1, AB908286.1, and AB908287.1) was propagated in 293T cells at an MOI of 0.001. The culture supernatants were harvested at full CPE and titrated using Vero cells with the standard TCID_50_ assay in a 96-well plate.

### 2.2. Viral RNA extraction

Step-by-step protocols for PCR-NGS and RCA-NGS methods were prepared as Supplementary documents 1 and 2, and has been deposited in the protocols.io repository (Misu et al., 2023a,b).

The working stock virus was centrifuged at 6,000 ×*g* for 10 min to remove debris. The ensuing supernatant was treated with Micrococcal Nuclease, a non-specific endo-exonuclease (catalog number M0247S, New England BioLabs, Ipswich, MA) to digest unwanted nucleic acids except for viral RNA. The reaction was performed in a final reaction volume of 200 μL, containing 180 μL of the supernatant 1 μL Micrococcal Nuclease and 20 μl of Micrococcal Nuclease Buffer (10X), and was incubated at 37°C for 1 h.

The RNA containing the viral genome was purified from the working stock virus treated with the Micrococcal Nuclease using a High Pure Viral RNA Kit (catalog number 11858882001, Roche Applied Science, Penzberg, Germany) according to the manufacturer’s instructions. The elution volume for RNA extraction was 50 μL.

Subsequently, the RNA sample containing primarily unwanted DNA derived from virus-infected cells was treated with a TURBO DNA-free Kit (catalog number AM1907, Thermo Fisher Scientific), which contains DNase I, an endonuclease capable of digesting both single- and double-stranded DNA. The reaction was performed in a final reaction volume of 56 μL containing the RNA sample, 1 μL DNase I and 5 μL of 10X reaction buffer. The reaction was conducted at 37°C for 30 min as per the manufacturer’s instruction, followed by the RNA concentration using the NucleoSpin RNA Clean-up XS Kit (catalog number 740903.10, Takara Bio). The purified RNA was collected in an elution volume of 10 μL.

### 2.3. Linker ligation

Purified viral RNA was ligated at the 3′ terminal with cSP6-polyA linker DNA (cSP6-L), which was synthesized by Integrated DNA Technologies (Coralville, IA) (Table 1) using T4 RNA Ligase 2, truncated KQ (catalog number M0373S, New England BioLabs). The reaction was performed in a final reaction volume of 20 μL containing 10 μL of the purified RNA, 1 μL of 10 μM cSP6-L, 2 μL of 10X T4 RNA Ligase Reaction Buffer, 6 μL of 50% PEG 8000 solution and 1 μL of T4 RNA Ligase 2, truncated KQ. The reaction was conducted at 25°C for 15 min according to the manufacturer’s instructions. cSP6-L consists of pre-adenylated at the 5′ terminal (5rApp), the complementary sequence of SP6 (Table 1), oligo (dA) 20 and dideoxycytidine (3ddC) at the 3′ terminal. Note that the SP6 sequence serves as the barcode to identify the true end of the viral terminal sequence. The reaction mixture was buffer-changed and concentrated to 10 μL of RNase-free water using the NucleoSpin RNA Clean-up XS Kit according to the manufacturer’s instructions.

**Table 1:** List of linkers and primers used in this study.

Name	Abbreviation	Type	Sequence (5′ to 3′)^a^
cSP6-polyA linker DNA	cSP6-L	DNA	5rApp-CTATAGTGTCACCTAAATCAAAAAAAAAAAAAAAAAAAA-3ddC
5′ phosphorylated SP6 primer	SP6 primer	DNA	5phos-GATTTAGGTGACACTATAG
VN primer	VNP	DNA	5phos-ACTTGCCTGTCGCTCTATCTTCTTTTTTTTTTTTTTTTTTTTVN
Strand-switching primer	SSP	DNA	TTTCTGTTGGTGCTGATATTGCTmGmGmG
qPCR for β-actin mRNA	β-actin-mRNA-F	DNA	CCACCATGTACCCTGGCATT
	β-actin-mRNA-R	DNA	CAGACTCGTCATACTCCTGC
	β-actin-mRNA-probe	DNA	FAM-GCCCTGGCGCCCAGCACGAT-BHQ1
qPCR for LCMV NP RNA	LCMV-NP-F	DNA	ATGCAGTCCAWRAGYGCA
	LCMV-NP-R	DNA	TATGARGACAAAGTNTGGGACAA
	LCMV-NP-probe	DNA	FAM-TTGTCTCTCACTACCCCAGTGTGCAT-BHQ1
qPCR for β-actin in genomic DNA	β-actin-genome-F	DNA	GTGCTGTCCCTGTACGCCTC
	β-actin-genome-R	DNA	GGCCATCTCCTGCTCGAAGT
	β-actin-genome-probe	DNA	FAM-GCGCGGCTACAGCTTCACCA-BHQ1
MI-R5’ Linker RNA		RNA	AUCGUAGGCACCUGACC

^a^5rApp, pre-adenylated at the 5’ terminal; 3ddC, dideoxycytidine at the 3′ terminal; 5phos, phosphorylated at the 5′ terminal; mG, 2-O-methylguanine.

### 2.4. RT and cDNA amplification by PCR

The cSP6-L-ligated viral RNA was reverse transcribed using Maxima H Minus Reverse Transcriptase (catalog number EP0751, Thermo Fisher Scientific) according to the cDNA-PCR Sequencing protocol (protocol version: PCSB_9086_v109_revK_14Aug2019) provided by Oxford Nanopore Technologies. Briefly, 9 μL of the purified RNA was combined with 1 μL of 10 mM dNTP mix and 1 μl of the VN primer (Table 1) supplied in a cDNA-PCR Sequencing Kit (catalog number SQK-PCS109, Oxford Nanopore Technologies) to yield a final volume of 11 μL, which was incubated at 65°C for 5 min and 4°C for 1 min. Subsequently, 11 μL sample was mixed with 4 μL of 5X RT buffer, 1 μL of SUPERase-In RNase inhibitor (catalog number AM2694, Thermo Fisher Scientific), 1 μL of H_2_O and 2 μL of strand-switching primer (SSP) (Table 1) supplied in the cDNA-PCR Sequencing kit (catalog number SQK-PCS109, Oxford Nanopore Technologies) to yield a final volume of 19 μL at 42°C, which was incubated for 2 min. Finally, 19 μL sample was combined with 1 μL of Maxima H Minus Reverse Transcriptase, resulting in a final volume of 20 μL, which was incubated at 42°C for 1.5 h and 85°C for 5 min.

The cDNA was amplified using PCR in a final reaction volume of 100 μL containing 5 μL of cDNA, 50 μL of KOD One PCR Master Mix (catalog number KMM-101, TOYOBO CO. LTD., Osaka, Japan), 3 μL of rapid barcoding primer (LWB) supplied in the PCR Barcoding Kit (catalog number SQK-PBK004, Oxford Nanopore Technologies), and 42 μL of H_2_O. PCR was performed with an initial denaturation step at 98°C for 15 s, followed by 30 cycles of amplification at the following conditions: 98°C for 10 s, 62°C for 5 s, and 68°C for 35 s (for LCMV, SFTSV, IAV and PRV), 75 s (for CDV) or 150 s (for SARS-SoV-2) at 5 s/kb, followed by a final extension of 68°C for 2 min.

Alternatively, Q5 Hot Start High-Fidelity 2X Master Mix (catalog number M0494S, New England BioLabs) was used to determine the whole genome sequences of CDV and SARS-CoV-2 instead of KOD One PCR master mix while maintaining the reaction volume. PCR was performed with an initial denaturation step at 98°C for 30 s, followed by 30 cycles of amplification under the following conditions: 98°C for 10 s and 72°C for 10 s and 72°C for 10 min (for CDV) or 20 min (for SARS-CoV-2) at 40 s/ kb, followed by a final extension of 72°C for 2 min.

The residual PCR primers in the reaction (100 μL) were eliminated by the addition of 2 μL of Exonuclease I (catalog number M0293S, New England BioLabs), a single-stranded DNA-specific exonuclease, and subjected to incubation at 37°C for 15 min and 80°C for 15 min. The purification of PCR product was achieved by combining 80 μL (a 0.8-fold volume) of AMPure XP Reagent (catalog number A63880, Beckman Coulter, Brea, CA) with 102 μL of PCR product. The purification process was completed by washing the mixture three times with 200 μL of 70% ethanol and eluted with 12 μL of elution buffer, which was supplied with the cDNA-PCR Sequencing Kit (SQK-PCS109, Oxford Nanopore Technologies).

The concentration of the purified cDNA was quantified using a Qubit 4 Fluorometer (Thermo Fisher Scientific) and Qubit 1X dsDNA High Sensitivity (HS) Assay Kit (catalog number Q33230, Thermo Fisher Scientific) according to the manufacturer’s instruction. The molar quantity of cDNA in the sample was converted from the concentration through the utilization of the viral genome length or the mean viral genome length if the viral genome is segmented.

Then, fifty to one hundred fmol of the purified sample (11 μL) were ligated with 1 μL of the Rapid adapter (RAP) supplied in the cDNA-PCR Sequencing kit or the PCR Barcoding Kit in a reaction volume of 12 μL at room temperature for 5 min. The resulting ligated sample was used for loading into the MinION flow cell as per manufacturer’s instruction.

### 2.5. RT and cDNA amplification by rolling circle amplification

The cSP6-L-ligated viral RNA was reverse transcribed using SuperScript IV Reverse Transcriptase (catalog number 18090050, Thermo Fisher Scientific). Briefly, 10 μL of the purified RNA was treated with 1 μL of 10 mM dNTP mix, 1 μL of H2O and 1 μL of 50 μM 5′ phosphorylated SP6 primer (Table 1), which is complementary to the sequence of the cSP6 linker DNA in a final volume of 13 μL, and subjected to incubation at 65°C for 5 min and 4°C for 1 min. Thereafter, the sample was further incubated with the addition of 4 μL of 5X SSIV buffer, 1 μL of SuperScript IV Reverse Transcriptase, 1 μL of 100 mM DTT and 1 μL of SUPERase-In RNase inhibitor in a total reaction volume of 20 μL at 55°C for 10 min, and then at 80°C for 10 min. Finally, the RNA in the RT reaction mix was digested with the addition of 1 μL of RNase H (catalog number M0297S, New England BioLabs) at 37°C for 20 min.

The cDNA present in the 21 μL of RT reaction mixture underwent ethanol precipitation, achieved through the addition of 20 μL of TE buffer (pH8.0), 4 μL of 3 M sodium acetate (pH5.2), 1 μL of Dr. GenTLE Precipitation Carrier (catalog number 9094, Takara Bio) and 100 μL of 100% ethanol. Subsequently, the mixture was centrifuged at 13,000 ×*g* for 15 min, yielding a precipitate. The pellet was then dissolved in 12 μL of H_2_O.

The cDNA was circularized using CircLigase II ssDNA Ligase (catalog number CL9021K, Biosearch Technologies, Hoddesdon, United Kingdom). The reaction was performed in a final reaction volume of 20 μL containing 12 μL of the cDNA, 2 μL of 10X reaction buffer, 1 μL of 50 mM MnCl_2_, 4 μL of 5 M Betaine and 1 μL of CircLigase II. The reaction mixture was incubated at 60°C for 1 h and 80°C for 10 min according to the manufacturer’s instructions.

The circularized cDNA solution (20 μL) underwent buffer exchange to H_2_O (10 μL) through ethanol precipitation according to the protocol described above and was subsequently amplified by rolling circle amplification (RCA) using Illustra Ready-To-Go GenomiPhi V3 DNA Amplification Kit (catalog number 25-6601-24, Cytiva, Tokyo, Japan) as per the manufacturer’s instructions, except for extending the reaction time from 1.5 h to 4 h. The cDNA solution (10 μL) was initially combined with 2X denaturation buffer (10 μL), incubated at 95°C for 3 min, and then chilled at 4°C. A ready to go Genomiphi cake was added to the denatured cDNA (20 μL), followed by incubation at 30°C for 4 h and then at 65°C for 10 min. The amplified cDNA was purified by adding 36 μL (a 1.8-fold volume) of AMPure XP regent, washing twice with 200 μL of 70% ethanol, and eluting with 40 μL of H_2_O. The total amplified cDNA was determined to be over 1,500 ng, as confirmed using a Qubit 4 Fluorometer and Qubit 1X dsDNA HS Assay Kit.

The following procedure, which involved branching, end-prepping and ligating sequencing adapters to the amplified cDNA, was modified from the protocols of the premium whole genome amplification protocol (version: WAL_9070_v109_revQ_14Aug2019) and Native Barcoding genomic DNA (NBE_9065_v109_revAK_14Aug2019) using a ligation sequence kit (SQK-LSK109), Native Barcoding Expansion 1-12 and 12-24 (EXP-NBD104 and EXP-NBD114) supplied by the Oxford Nanopore Technologies.

The amplified cDNA was digested using T7 endonuclease I (catalog number M0302S, New England BioLabs), to remove the branching. The reaction was performed in a final reaction volume of 30 μL containing 1 μg of cDNA, 3 μL of NEBuffer 2, 1.5 μL of T7 endonuclease I, and H_2_O. The reaction mixture was incubated at 37°C for 30 min. The cDNA was then purified by adding 54 μL (a 1.8-fold volume) of AMPure XP Reagent, washing twice with 200 μL of 70% ethanol, and eluting with 24 μL of H_2_O.

The purified cDNA was repaired and end-prepped using NEBNext FFPE DNA Repair Mix (catalog number M6630, New England BioLabs) and NEBNext Ultra II End Repair/dA-Tailing Module (catalog number E7546, New England BioLabs). The reaction was performed in a final reaction volume of 30 μL containing 24 μL of cDNA, 1 μL of NEB Next FFPE DNA Repair Mix, 1.75 μL of NEB Next FFPE DNA repair buffer, 1.5 μL of NEBNext Ultra II end-prep enzyme Mix, 1.75 μL of NEBNext Ultra II end-prep reaction buffer. The reaction mixture was incubated at 20°C for 30 min and then 65°C for 5 min. The cDNA was then purified by adding 54 μL (a 1.8-fold volume) of AMPure XP Reagent, followed by two washes with 200 μL of 70% ethanol and elution with 30 μL of H2O. The total amount of cDNA was approximately 700 ng or more, as confirmed using a Qubit 4 Fluorometer and Qubit 1X dsDNA HS Assay Kit.

The purified cDNA was ligated with Native Barcoding Expansion (catalog number EXP-NBD104 and EXP-NBD114, Oxford Nanopore Technologies). The reaction was performed in a final reaction volume of 25 μL containing 400 ng of cDNA, 12.5 μL of Blunt/TA ligase master mix (catalog number M0367S, New England BioLabs), 1.5 μL of Native Barcoding Expansion and H_2_O. The reaction mixture was incubated at 25°C for 20 min and then mixed with an equal volume (25 μL) of TE (pH8.0) before purification. The purification procedure involved the addition of 40 μL (a 0.8-fold volume) of AMPure XP Reagent, followed by two washings with 200 μL of 70% ethanol and elution with 20 μL of H_2_O. The purified cDNA concentration was determined using a Qubit 4 Fluorometer with a Qubit 1X dsDNA HS Assay Kit. The molar concentration of the cDNA sample was calculated based on the length of the major band confirmed by electrophoresis after T7 endonuclease treatment, which typically ranges around 2000 base pairs.

The cDNA was ligated with sequencing adaptors using NEBNext Quick Ligation Module (catalog number E6056S, New England BioLabs) in a final reaction volume of 50 μL. The reaction comprised 100–200 fmol of cDNA, 2.5 μL of Adaptor Mix II supplied in the Native Barcoding Expansion, 10 μL of NEB Next Quick Ligation Reaction Buffer (5X), 5 μL of Quick T4 DNA ligase and H_2_O followed by incubation at 25°C for 20 min. The adaptor-ligated cDNA was purified by adding 80 μL of AMPure XP Reagent, followed by two washes with 200 μL of Short Fragment Buffer (SFB), and eluting with 12 μL of Elution Buffer (EB) supplied in the ligation sequence kit.

The concentration of the eluted cDNA was quantified using a Qubit 4 Fluorometer with a Qubit 1X dsDNA HS Assay Kit. A 50–100 fmol sample was used for loading into the MinION flow cell.

### 2.6. Short cDNA fragment removal before RCA

PRV RNA purification, cDNA synthesis, and RNA digestion using RNase H were performed as described above. Subsequently, instead of ethanol precipitation, cDNA was purified using AMPure XP Reagent. The cDNA (21 μL) was mixed with 36 μL (a 1.8-fold volume) of AMPure XP Reagent, which was then washed twice with 200 μL of 70% ethanol and eluted with 20 μL of TE buffer. Moreover, the eluted cDNA was mixed with 16 μL (x 0.8 vol.) of AMPure XP Reagent and then washed with 200 μL of 70% ethanol twice and eluted with 20 μL of TE buffer to remove short cDNA fragments (<200 bp). The subsequent steps involved circularization and other procedures, as described above.

### 2.7. Quantitative PCR

One-step reverse transcription-qPCR (RT-qPCR) or quantitative PCR (qPCR) was utilized to evaluate the efficacies of nucleases treatment and the cSP6-L ligation.

To determine the LCMV NP RNA copies within the purified viral RNA sample, 2 μL of the sample was added to the reaction mixture for the QuantiTect probe RT-PCR kit (catalog number 204343, Qiagen, Hilden, Germany), which comprised of 2X QuantiTect probe PCR master mix, QuantiTect RT mix, H_2_O, specific primers and probe for LCMV NP RNA (sequences of the primers and the probe named LCMV-NP-F, −R and -probe are shown in Table 1) at a concentration of 4 μM of each primer and 2 μM of probe. RT-qPCR cycling was performed using a LightCycler 96 (Roche) under the following conditions: the reverse transcription reaction was carried out at 50°C for 30 min, 95°C for 15 min, and then 45 cycles of 95°C for 15 s, 60°C for 1 min.

Specifically, to evaluate the ligation efficacy of cSP6-L, the copy number of LCMV NP RNA was quantified by two-step RT-qPCR. According to the aforementioned protocol, the cSP6-L ligated LCMV RNA solutions were reverse transcribed utilizing SuperScript IV Reverse Transcriptase with SP6 primer (Table 1). As an input control, the LCMV RNA solutions without cSP6-L ligation were reverse transcribed using a random hexamer primer. For the qPCR assay, 2 μL of the cDNA solution was added to the reaction mixture for the QuantiTect probe RT-PCR kit without the addition of the reverse transcriptase solution (i.e., QuantiTect RT mix). The reaction mixture comprised of 2X QuantiTect probe PCR master mix, H_2_O, specific primers and probe to LCMV NP RNA (LCMV-NP-F, -R and -probe) at a concentration of 4 μM of each primer and 2 μM of the probe. qPCR amplification was performed using a LightCycler 96 (Roche) under the following conditions: 45 cycles of 94°C for 15 s and 60°C for 60 s, followed by PCR activation at 95°C for 15 min.

To determine DNA contamination in the purified viral RNA sample derived from LCMV-infected Vero cells, the copy number of the green monkey β-actin region in genomic DNA was quantified. For the qPCR assay, 2 μL of the sample was added to the reaction mixture for the QuantiTect probe RT-PCR kit without adding the reverse transcriptase solution. The reaction mixture consisted of 2X QuantiTect probe PCR master mix, H_2_O, specific primers and probe to β-actin in genomic DNA (sequences of the primers and the probe named β-actin-genome-F, -R and -probe are shown in Table 1) at a concentration of 4 μM of each primer and 2 μM of the probe. qPCR amplification was performed using a LightCycler 96 (Roche) under the following conditions: 45 cycles of 94°C for 15 s and 60°C for 60 s, followed by PCR activation at 95°C for 15 min.

To evaluate mRNA contamination in the purified viral RNA sample derived from LCMV-infected Vero cells, the copy number of the green monkey β-actin mRNA was quantified. For the RT-qPCR assay, 2 μL of the sample was added to the reaction mixture for the QuantiTect probe RT-PCR kit, which consisted of 2X QuantiTect probe PCR master mix, QuantiTect RT mix, H_2_O, and specific intron-spanning primers and probe (sequences of primers and prove named β-actin-mRNA-F, -R and -probe are shown in Table 1) at a concentration of 4 μM of each primer and 2 μM of the probe. RT-qPCR cycling was performed using a LightCycler 96 (Roche) under the following conditions: the reverse transcription reaction was carried out at 50°C for 30 min, 95°C for 15 min, and then 45 cycles of 95°C for 15 s, 60°C for 1 min.

### 2.8. qPCR standard preparation

LCMV RNA reference was generated using a MEGAscript T7 kit (catalog number AM1333, Thermo Fisher Scientific) from LCMV NP gene DNA (position 1,713–2,238 in Genbank accession number JF912085) that was synthesized by Integrated DNA Technologies. The synthesized DNA with the T7 promoter sequence at the 5′ end was added. A plasmid containing part of the β-actin mRNA sequence (position 9–1,137 in Genbank accession number NM_001330273) was utilized as a reference for β-actin mRNA and genomic DNA.

### 2.9. NGS and reference alignment

The samples were sequenced by a next-generation sequencer MinION Mk1B (Oxford Nanopore Technologies) with a flow cell R9.4 (catalog number FLO-MIN106, Oxford Nanopore Technologies), and the sequencing data was basecalled with Guppy (v4.2.2) fast mode. Even when the samples were multiplexed on a single flow cell using Native Barcoding Expansion, the sequencing results were divided into separate FASTQ formatted files for each sample.

The reference viral genome sequences were prepared with the addition of the SP6 primer sequence (Table 1) and the SSP sequence (Table 1) at the 3′ and 5′ ends, respectively, when PCR amplified the cDNA samples (PCR-NGS method).Otherwise, these were prepared with the addition of the SP6 primer sequence at both the 3′ and 5′ ends when amplifying cDNA samples through RCA (RCA-NGS method).

The NGS reads were aligned to the reference viral genome using the NanoPipe web service (Shabardina et al., 2019), with the default settings. The read depth aligned to viral reference genomes was extracted from BAM and indexed BAM files generated by NanoPipe using the SAMtools software program (Li et al., 2009).

### 2.10. Sanger sequencing

Purified RNA from the working stocks of SFTSV, IAV, CDV were subjected to Sanger sequencing when it was necessary to determine some viral genome sequences. First, RT was performed using SuperScript IV Reverse Transcriptase with random hexamer primer according to the manufacturer’s instructions. Then the cDNA was amplified using KOD One PCR Master Mix with the specific primer sets for the viral genome sequences. Next, the PCR products were sequenced using a BigDye Terminator v3.1 Cycle Sequencing Kit (catalog number 4337456, Applied Biosystems) and Applied Biosystems ABI3130sequencer 3130XL Genetic Analyzer.

In addition, the terminal sequences of the IAV genomes were determined by rapid amplification of cDNA ends (RACE) at the 3′ and 5′ ends with Sanger sequencing. Briefly, 10 μL of purified RNA from the working stock of IAV was ligated at its 3′ end with the cSP6-L described above. Next, the reaction was purified using NucleoSpin RNA Clean-up XS Kit with a final elution volume of 10 μL of RNase-free water. Next, the 10 μL of purified RNA was ligated its 5′ end with 1 μL of 10 μM MI-R5’ Linker RNA (Table 1) (catalog number DS330, Biodynamics Laboratory, Tokyo, Japan) using T4 RNA Ligase 1 (catalog number M0204S, New England BioLabs) in a final reaction volume of 25 μL at room temperature for 15 min. According to the manufacturer’s instructions, the purified RNA was reverse transcribed using SuperScript IV Reverse Transcriptase with a random hexamer primer. Then the cDNA was amplified using KOD One PCR Master Mix with the 3′ or 5′ linker-specific primer and virus-specific primers. Finally, the PCR products were subjected to Sanger sequencing.

### 2.11. Taxonomic profiling

Based on the default setting, the total FASTQ reads were analyzed using blastn for NT database (SE) on the Maser data analysis platform (Kinjo et al., 2018). Otherwise, the unmapped FASTQ reads extracted from the reference-aligned BAM files generated by the BWA-MEM sequence aligner were analyzed using blastn for NT database (SE) on the Maser platform. Finally, the blastn results were taxonomically classified using the MEGAN software program (Huson et al., 2018) based on the default setting, except for changing the top percentage from 10.0 to 0.5.

### 2.12. Detection of mycoplasma contamination

According to the manufacturer’s instructions, mycoplasma detection in the working virus stocks was performed using the Takara PCR Mycoplasma detection set (catalog number 6601, Takara bio). The kit can detect at least 11 species of *Mycoplasma* (i.e., *M. fermentans, M. hyorhinis, M. arginini, M. orale, M. salivarium, M. hominis, M. pulmonis, M. arthritidis, M. neurolyticum, M. hyopneumoniae, M. capricolum*) and species of *Ureaplasma* (i.e., *U. urealyticum*) belonging Mycoplasmataceae.

### 2.13. Statistical analysis

All statistical analyzes were performed using GraphPad Prism 9 (GraphPad Software, La Jolla, CA). The mean LCMV NP copy numbers and cDNA yields amplified by RCA were compared using a two-way analysis of variance (ANOVA) with Dunnett’s multiple-comparison test or an unpaired *t*-test. value of *p* < 0.05 were considered to indicate statistical significance.

## 3. Results

### 3.1. Summary of the NGS methods

**Figure 1:**
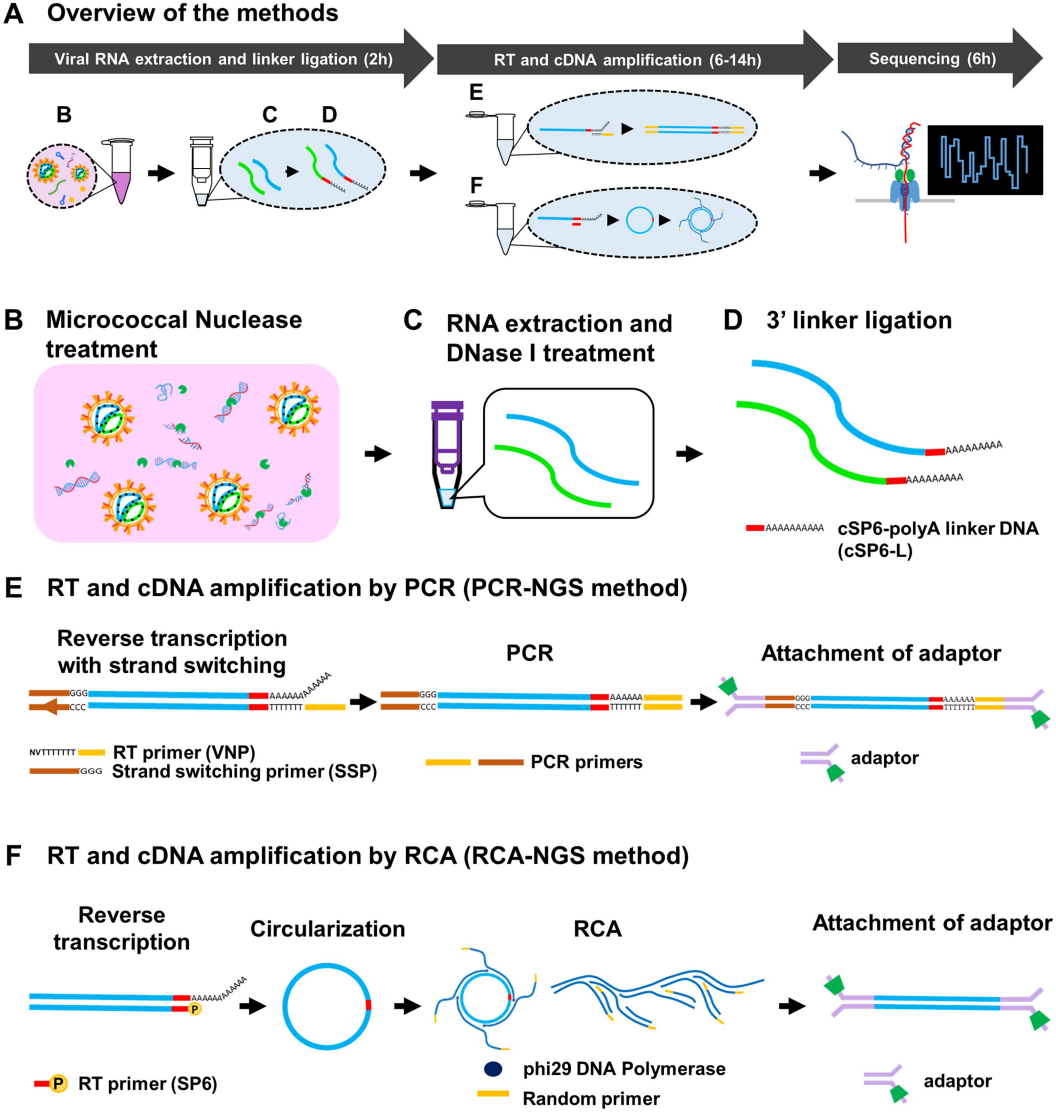
Schematic diagram of the methods for determination of entire RNA viral genome. Overview of the methods and approximate working hours **(A)** can be divided roughly into viral RNA extraction and linker ligation, RT and cDNA amplification by PCR or RCA, and Sequencing. The Micrococcal Nuclease, which is derived from *Staphylococcus aureus* and is a non-specific endo-exonuclease, treatment **(B)** to digest both DNA and RNA except viral RNA, DNase I treatment **(C)** to digest remaining DNA and 3′ barcoded polyA linker DNA (cSP6-L) ligation **(D)** were performed at the virus RNA extraction and linker ligation step. At the RT and cDNA amplification step, RNA in the sample is reverse transcribed using poly(T)-containing anchored RT primer (VNP) with strand-switching to add a unique sequence of SSP at the 5′ end and amplified by PCR **(E)**. Otherwise, the RNA is reverse transcribed using 5′-phosphorylated SP6 primer (SP6), circularized using CircLigase II, and amplified by RCA using phi29 DNA polymerase with random primers **(F)**. These amplified cDNAs are then attached to the adaptors for sequencing. Note that the SP6 sequence serves as the barcode to identify the true end of viral terminal sequence.

We established two methods, PCR-NGS and RCA-NGS, that enable whole RNA viral genome sequencing regardless of the genomic strandness. The essentials of either method are to purify only the viral RNA and amplify only the viral cDNA. These methods for preparing cDNA samples can be divided into three parts: (1) viral RNA extraction and linker ligation; (2) RT and cDNA amplification; and (3) sequencing using MinION (Figure 1A). In the first part, culture supernatant from cells infected with an RNA virus (i.e., a working stock of an RNA virus) is used. The sample is treated with the Micrococcal Nuclease, a non-specific endo-exonuclease to digest unwanted nucleic acids (i.e., DNA and RNA molecules mainly derived from the virus-infected cells) except for viral RNA, which is protected from the Micrococcal Nuclease by its envelope or capsid (Figure 1B). Following RNA extraction, the purified RNA is treated with DNase I to digest the remaining DNA (Figure 1C). Then the RNA is ligated to the 3′ end with the barcoded polyA linker DNA (i.e., cSP6-L) (Figure 1D). The cSP6-L contains the specific sequence (i.e., the complementary sequence of SP6 primer) to identify the 3′ terminal viral genome sequence. The linker-ligated RNA is reverse transcribed and then amplified by either PCR or RCA without using the virus-specific primers.

To amplify the cDNA by PCR, named PCR-NGS method, RT is performed. The reverse transcriptase using the PCR-NGS method has a strand switching activity to ligate the strand switching primer (SSP) to the 5′ end of the RNA (Figure 1E). The SSP sequences guide the identification of the 5′ terminal sequence of the viral genome. On the other hand, to amplify viral cDNA by RCA, named RCA-NGS method, RT is performed using the 5′-phosphorylated primer. The phosphorylated primer is necessary for enzymatic cDNA circularizing and guiding both 3′ and 5′ terminal sequences of the viral genome (Figure 1F). The cDNA circularization is expected to amplify the 3′ and 5′ terminal sequences efficiently by phi29 DNA polymerase and provide sufficient read depth. After circularization, RCA is performed using phi29 DNA polymerase with a random primer. Following cDNA amplification by PCR or RCA, the sequencing adaptors are ligated, and the sequencing run is performed using MinION according to the manufacturer’s instructions.

### 3.2. Establishment of the NGS methods

**Figure 2:**
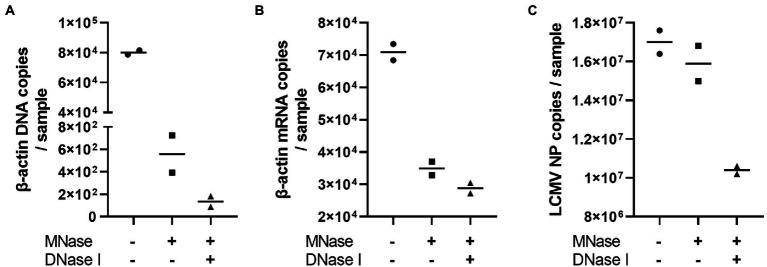
The effects of Micrococcal Nuclease and DNase I treatment. The LCMV culture supernatant was treated with DNase I for 30 min following Micrococcal Nuclease treatment for 60 min to prepare viral RNA. The residual genomic DNA, mRNA and LCMV RNA corresponding to the copy numbers of β-actin DNA **(A)**, β-actin mRNA **(B)** and LCMV NP RNA **(C)** in purified RNA solutions were measured after Micrococcal Nuclease (MNase) with or without DNase I (DNase I) treatments.

The problem with using the NGS technique for whole RNA viral genome sequencing is that the DNA and RNA contaminations originated from the virus-infected cells. Therefore, we attempted to optimize the PCR-NGS and RCA-NGS methods to amplify only viral genomic RNA while removing the cell-derived nucleic acids in the sample. The efficacy of Micrococcal Nuclease and DNase I treatment to remove unwanted RNA and DNA was first evaluated to establish the method. LCMV-infected Vero cell culture supernatant was treated with or without these nucleases according to the protocol established in this study. After Micrococcal Nuclease and DNase I treatment, unnecessary DNA and RNA contamination was estimated by measuring the copy numbers of LCMV NP RNA, monkey β-actin DNA, and mRNA (Figure 2). Micrococcal Nuclease treatment caused a dramatic approximately 100-fold reduction in the β-actin DNA copy numbers (Figure 2A) and a slight reduction in β-actin mRNA copy numbers (Figure 2B) with little effect in the LCMV NP copy number (Figure 2C) in comparison with those of without the Micrococcal Nuclease treatment. In contrast, DNase I treatment with Micrococcal Nuclease caused a four-fold reduction in the β-actin DNA copy numbers and little reduction in β-actin mRNA copy numbers with a slight but negligible reduction in the LCMV NP copy number (Figures 2A–C). These results indicated that Micrococcal Nuclease treatment without DNase I effectively reduced the amount of unwanted DNA and RNA in the sample without affecting the viral RNA.

**Figure 3:**
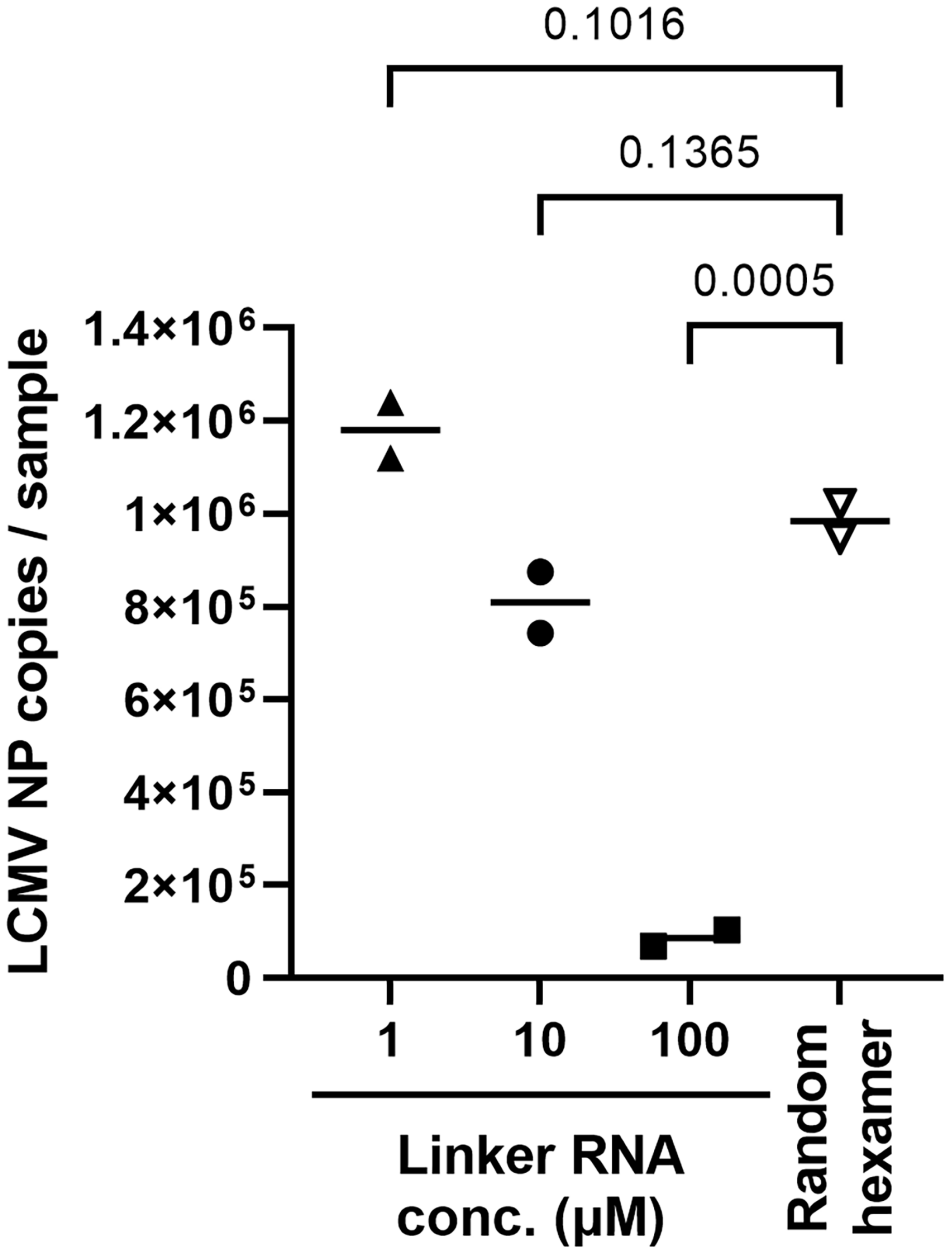
Ligation efficacy of cSP6-L at various linker concentrations. Purified RNA samples of LCMV were ligated with cSP6-L at various concentrations. First, RT was performed using SP6 primer following linker ligation. Subsequently, qPCR was performed to measure the copies of linker ligated LCMV NP cDNA in duplicate samples. Then, the same amount of purified RNA as the ligated cSP6-L was reverse-transcribed with random hexamer primers, and the copy number was measured for the experimental control. A two-way analysis of variance (ANOVA) with Dunnett’s multiple-comparison test was used to determine the level of statistical significance. The calculated value of ps are shown above the groups that were compared.

Next, cSP6-L concentration was optimized for the best linker-ligation efficacy. Total RNA was purified from LCMV-infected cell culture supernatant following Micrococcal Nuclease treatment and was ligated to the 3′ end with 1 μL of 1, 10, or 100 μM of cSP6-L DNA (i.e., final concentration was 0.05, 0.5, or 5 μM per reaction). To determine whether LCMV NP RNA successfully ligated the linker DNA, an evaluation was performed by two-step qRT-PCR. RT was performed using SP6 primer. Otherwise, the same amount of total RNA without performing linker ligation was reverse transcribed with random hexamer primers, as an experimental control. The copy numbers of LCMV NP cDNA in the samples were then measured by qPCR using an LCMV NP-specific primer set (Figure 3). The polyA DNA linker-ligated LCMV NP cDNA copy numbers were significantly reduced in comparison to the experimental control when the linker DNA concentration was 100 μM. On the other hand, the number of LCMV NP cDNA copies was not significantly altered in comparison to the control when the linker DNA concentration was 1 μM or 10 μM. Thus, to consider the possibility of reducing the amount of linker DNA when the total RNA amount in the sample is excessive, a concentration of 10 μM was used in subsequent experiments.

**Figure 4:**
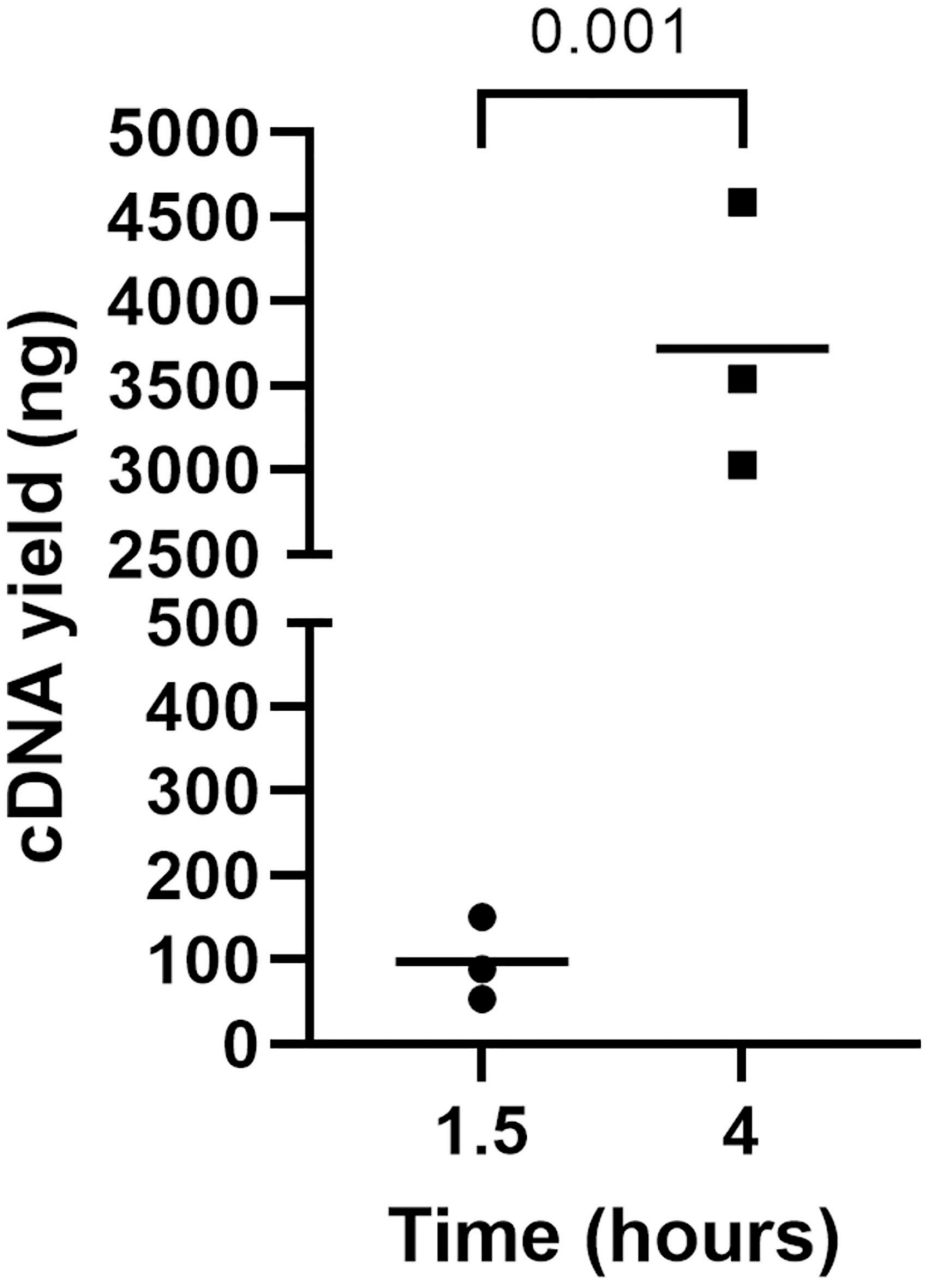
The impact of incubation time on cDNA yield by RCA. RCA amplified the circularized LCMV cDNA for 1, 5, or 4 h, and a Qubit 4 Fluorometer measured the yield of cDNAs. An unpaired *t*-test was used to determine the level of statistical significance. The calculated value of p are shown above the groups.

The incubation time of RCA was also optimized since the time indicated in the manufacturer’s instructions (i.e., 1.5 h) did not yield the required amount of RCA amplicons for NGS (i.e., 100–200 fmol) (Figure 4). Thus, circularized cDNA prepared according to an established method was amplified for 4 h and compared with circularized cDNA amplified for 1.5 h (Figure 4). The amount of RCA amplicons was approximately 100 ng when the reaction time was 1.5 h, whereas the amount significantly increased to around 3,500 ng when the reaction time was 4 h. Thus, the reaction time was fixed at 4 h for cDNA amplification by RCA.

### 3.3. Validation of the NGS methods

The established method was evaluated using RNA viruses, namely LCMV, SFTSV, and IAV, as negative-stranded viruses with segmented genomes, CDV as a negative-stranded virus with a non-segmented genome, PRV as a double-stranded virus with segmented genomes and SARS-CoV-2 as a positive-stranded virus, which carries the largest RNA genome (Table 2). The cDNA samples were prepared from 180 μL of working stock of RNA viruses that contained 5.4 × 10^4^–4.5 × 10^7^ TCID_50_ of the viruses (Table 2). Samples (100–200 fmol per flow cell) were prepared using the PCR-NGS or RCA-NGS method for the sequencing run.

**Table 2:** RNA viruses used in this study.

Virus	Abbreviation	Genome organisation^a^	Virion^b^	Number of segments	Genome size (bases)	Virus titer^b^ (TCID_50_/180 μL)
Lymphocytic choriomeningitis virus	LCMV	ss(−) RNA	enveloped	2	3.4–7.2 K	3.6 × 10^6^
Severe fever with thrombocytopenia syndrome virus	SFTSV	ss(−) RNA	enveloped	3	1.7–6.4 K	7.2 × 10^5^
Influenza A virus	IAV	ss(−) RNA	enveloped	8	0.89–2.3 K	4.5 × 10^7^
Canine distemper virus	CDV	ss(−) RNA	enveloped	non-segmented	15 K	5.4 × 10^4^
Severe acute respiratory syndrome coronavirus 2	SARS-CoV-2	ss(+) RNA	enveloped	non-segmented	30 K	5.9 × 10^5^
Pteropine orthoreovirus	PRV	ds RNA	nonenveloped	10	1.2–3.9 K	5.6 × 10^5^

^a^ss, single-stranded; ds, double-stranded; (−), negative-stranded; (+), positive-stranded. ^b^cDNA library samples were prepared from 180 μL of the working stock of viruses.

**Figure 5:**
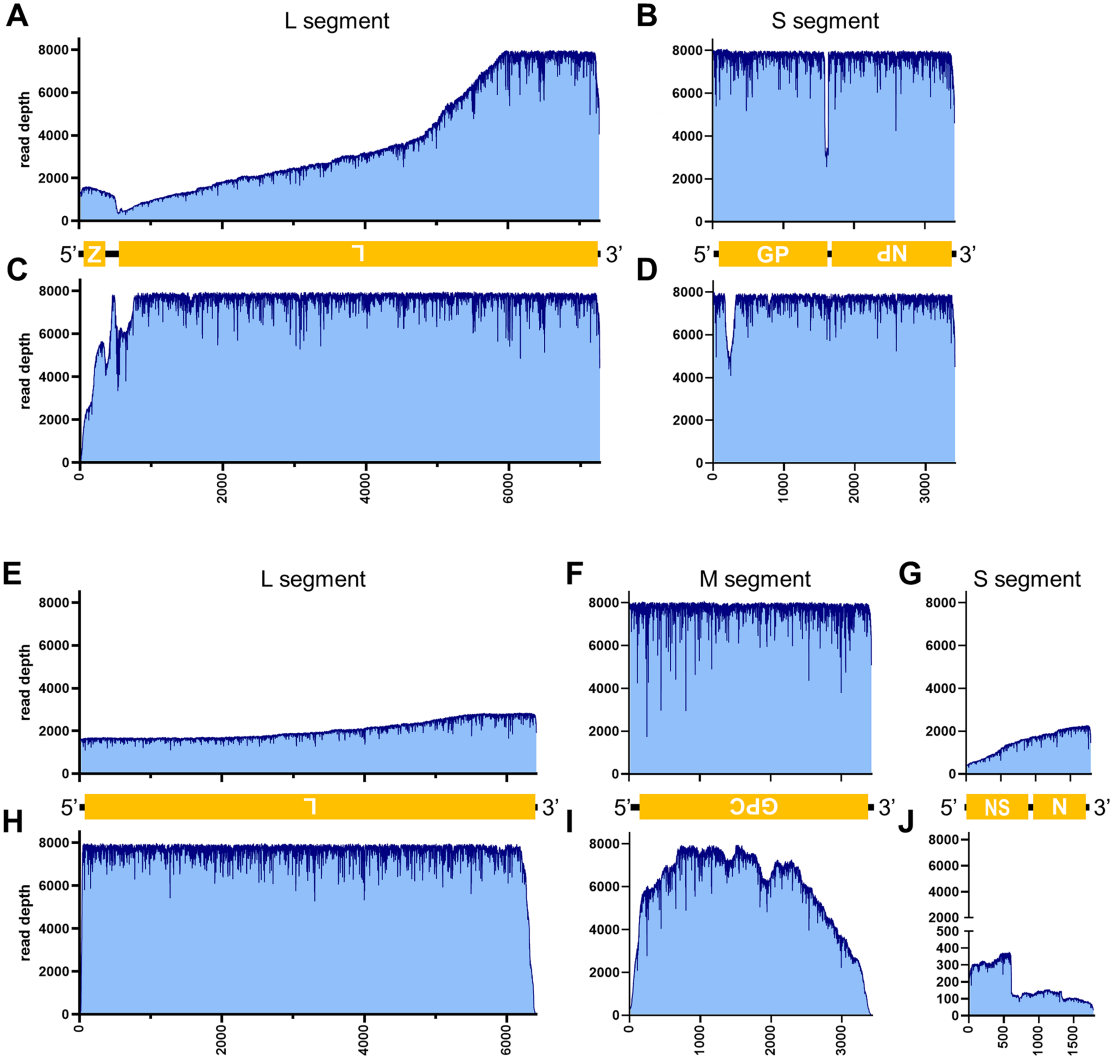
Read depth aligned to LCMV and SFTSV reference genomes. Read depth of LCMV **(A–D)**, and SFTSV **(E–J)** across the LCMV L **(A,C)** and S **(B,D)** segments and the SFTSV L **(E,H)**, M **(F,I)**, and S **(G,J)** segments using data generated from the PCR-NGS method [upper panels: **(A,B,E–G)**] or RCA-NGS method [lower panels: **(C,D,H–J)**] are shown. The schematic view of the open reading frames (ORFs) and UTRs are shown as yellow boxes and black lines in the 5′-to-3′ direction. The product of ORF is shown in the box, and the turned letter indicates the ORF is in the reverse complement (i.e., 3′-to-5′ direction). In addition, the number of reads (y-axis) obtained at each nucleotide base pair position (x-axis) is shown.

**Figure 6:**
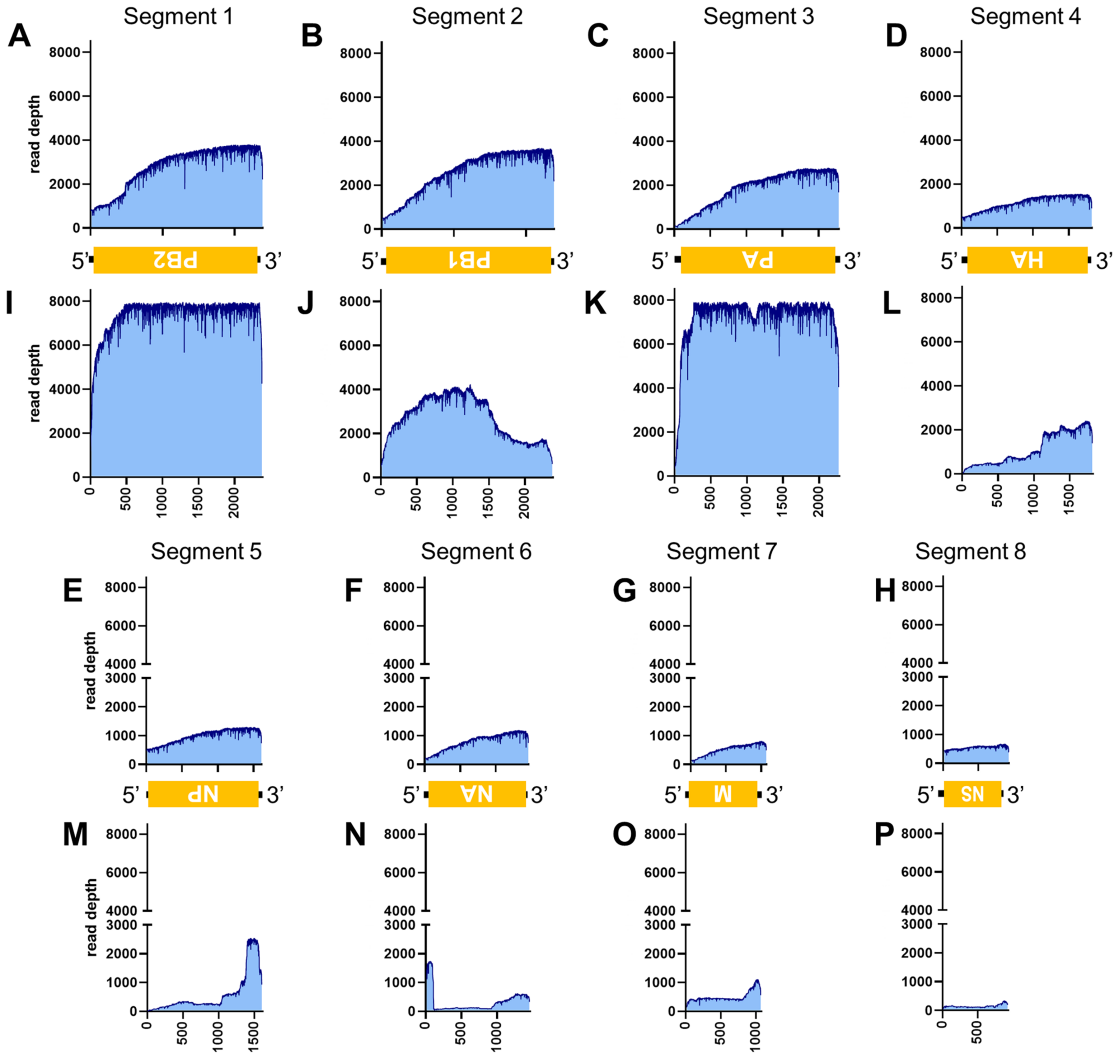
Read depth aligned to IAV reference genome. Read depth across the segments 1 **(A,I)**, 2 **(B,J)**, 3 **(C,K)**, 4 **(D,L)**, 5 **(E,M)**, 6 **(F,N)**, 7 **(G,O)** and 8 **(H,P)** segments using data generated from PCR-NGS method [upper panels: **(A–H)**] or RCA-NGS method [lower panels: **(I–P)**] are shown. The schematic view of the open reading frames (ORFs) and UTRs are shown as yellow boxes and black lines in the 5′-to-3′ direction. The product of ORF is shown in the box, and the turned letter indicates the ORF is in the reverse complement (i.e., 3′-to-5′ direction). The number of reads (y-axis) obtained at each nucleotide base pair position (x-axis) is shown.

**Figure 7:**
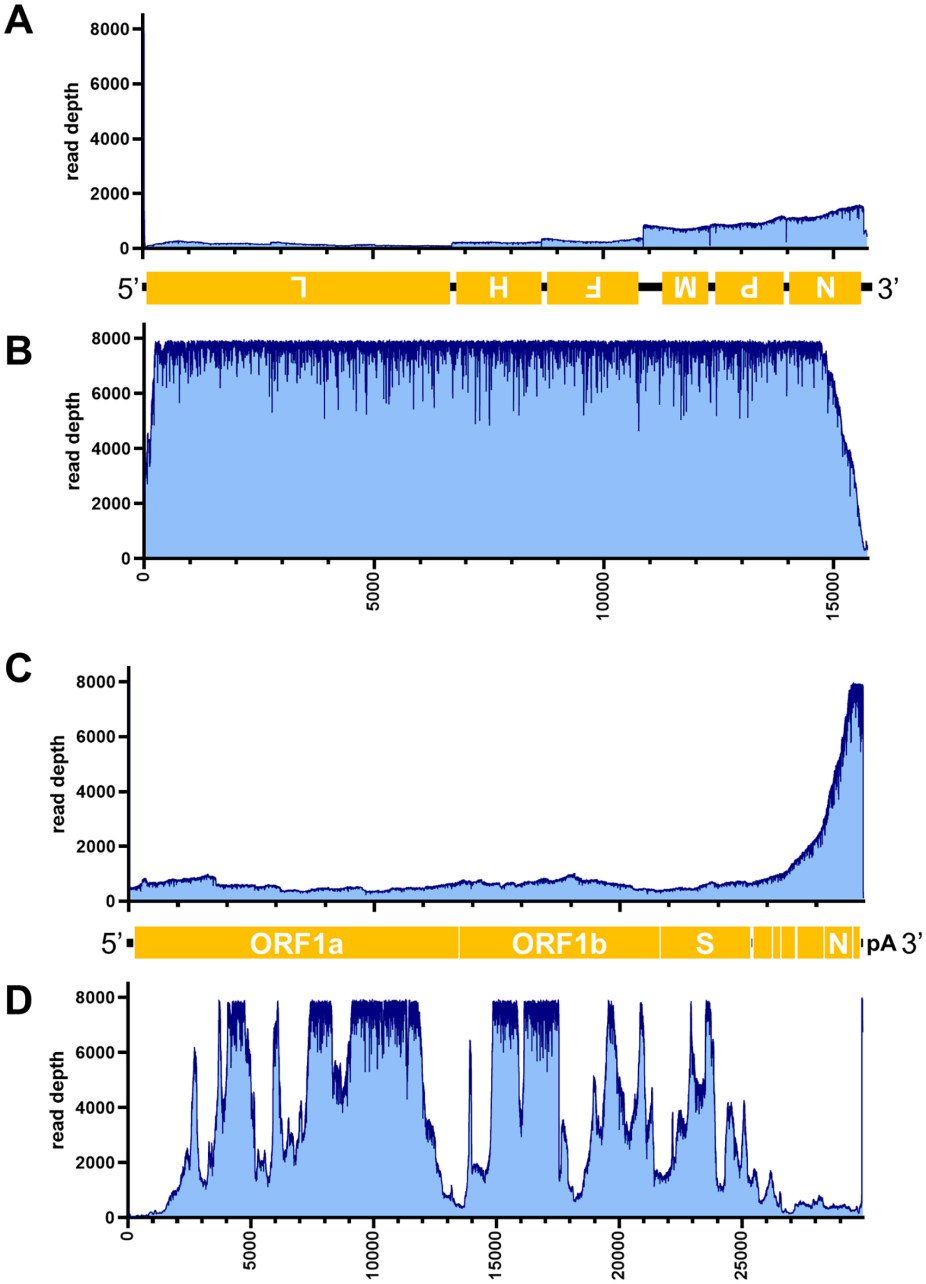
Read depth aligned to CDV and SARS-CoV-2 reference genomes. Read depth of CDV **(A,B)** and SARS-CoV-2 **(C,D)** across the genome using data generated from the PCR-NGS method [upper panels: **(A,C)**] or RCA-NGS method [lower panels: **(B,D)**] are shown. The schematic view of the open reading frames (ORFs) and UTRs are shown as yellow boxes and black lines in the 5′-to-3′ direction. The product of ORF is shown in the box, and the turned letter indicates the ORF is in the reverse complement (i.e., 3′-to-5′ direction). The number of reads (y-axis) obtained at each nucleotide base pair position (x-axis) is shown.

**Figure 8:**
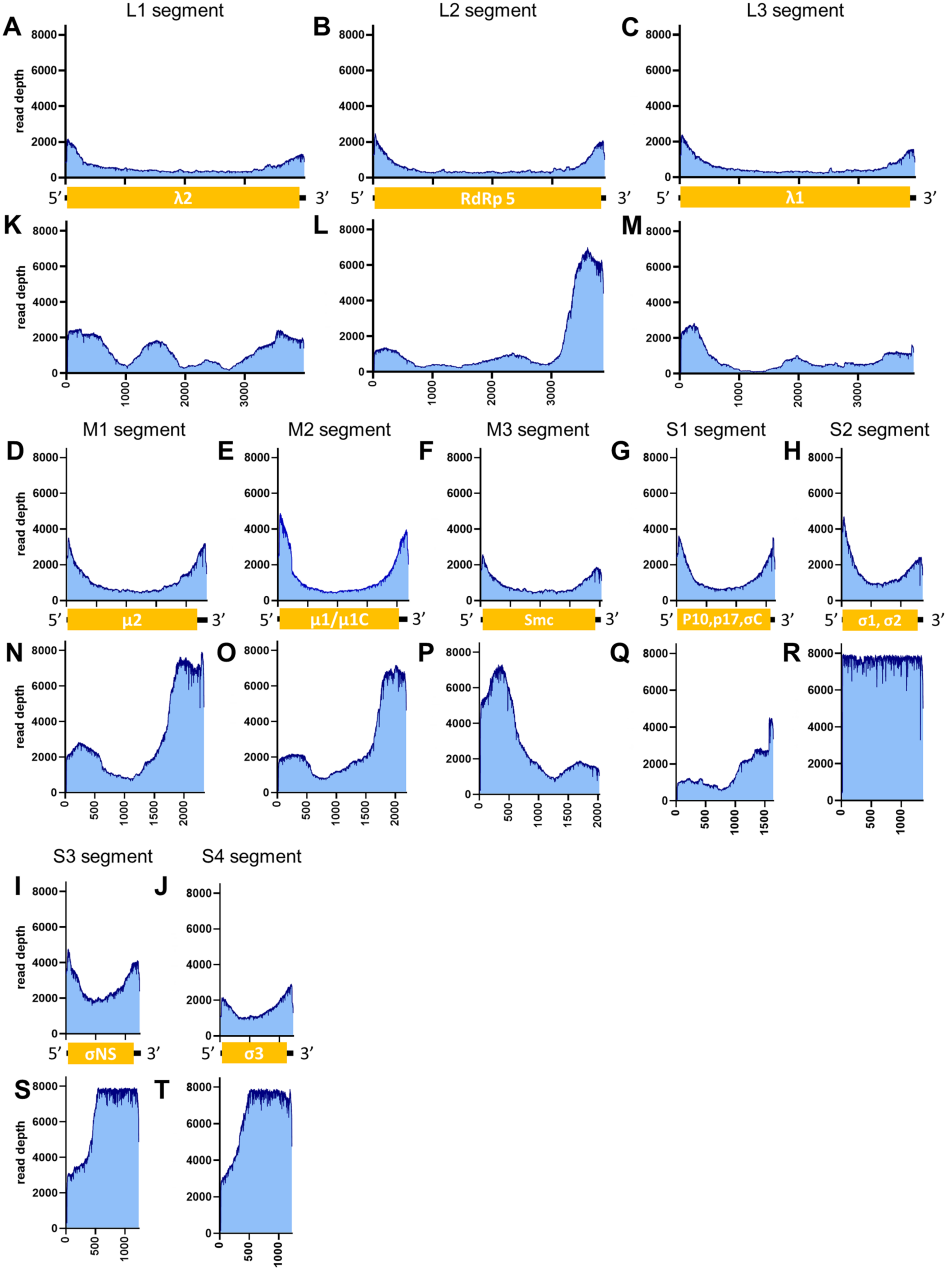
Read depth aligned to PRV reference genome. The data generated from the PCR-NGS method [upper panels: **(A–J)**] or RCA-NGS method [lower panels: **(K–T)**] aligned to the segments L1 **(A,K)**, L2 **(B,L)**, L3 **(C,M)**, M1 **(D,N)**, M2 **(E,O)**, M3 **(F,P)**, S1 **(G,Q)**, S2 **(H,R)**, S3 **(I,S)** and S4 **(J,T)** are shown. The number of reads (y-axis) obtained at each nucleotide base pair position (x-axis) is shown.

Table 3 illustrates the actual quantity of samples utilized per run, the total number of reads obtained from the run, the number of mapped reads, and the read depths analyzed using NanoPipe. The percent mapped reads varied from 2.1 to 99.5% (Table 3). The mean read depth of the samples (except for CDV and PRV determined by the PCR-NGS method) was more than 1,000× (Table 3). Upon the initial implementation of the PCR-NGS method using KOD One PCR master mix for the detection of CDV and SARS-CoV-2, the number of total reads was very small. The mapping of CDV comprised 53 mapped reads in proportion to 584 total reads, which were recorded 6 h post NGS run. Furthermore, the mapping of SARS-CoV-2 was even more limited, comprising only 4 mapped reads out of a total of 54 reads, recorded 6 h post NGS run. Therefore, the KOD One PCR master mix was substituted with the Q5 Hot Start High-Fidelity 2X Master Mix to achieve a more robust performance. Subsequently, the percentage of mapped reads improved to 2.1 and 16.7%, respectively (Table 3). For all samples, the breadth of coverage was 100%, and the coverage where the read depth was more than 100× was 95% or better. The read depths aligned to the LCMV, SFTSV, IAV, CDV, SARS-CoV-2 and PRV genomes were visualized from the results (Figures 5–8). The read depth obtained by the PCR-NGS method was characterized by a gradual decrease from the 3′ end to the 5′ end, whereas that obtained by RCA-NGS was not, probably because of the PCR processivity. In addition, the percentages of mapped reads of CDV and SARS-CoV-2 obtained using PCR-NGS were lower than those obtained by the RCA method (Table 3; Figure 7). The result indicates that large viral genomes (i.e., CDV: 15 k bases, SARS-CoV-2: 30 k bases) are more difficult to amplify by PCR than by RCA.

**Table 3:** NGS results.

Virus	Method	Amount used^a^		% Accuracy^c^	Number of reads^d^			Read depth		
		ng	fmol^b^		Mapped	Total	% Mapped	Mean^e^	Minimum^f^	% Coverage <100 reads^g^
LCMV	PCR-NGS	150	50	100	77,515	168,316	46.1	4,948	301	0
	RCA-NGS	115	40	100	318,119	732,446	43.4	4,564	256	0
SFTSV	PCR-NGS	236	65	100	18,921	20,977	90.2	3,577	313	0
	RCA-NGS	242	130	100	281,966	283,303	99.5	4,578	45	2.8
IAV	PCR-NGS	200	95	100	18,270	24,760	73.8	1,667	54	0.1
	RCA-NGS	255	194	100	62,960	68,287	92.2	3,142	27	3.9
CDV	PCR-NGS	256	25	100	9,199	442,272	2.1	451	69	4.6
	RCA-NGS	908	98	100	396,825	505,295	78.5	7,459	181	0
SARS-CoV-2	PCR-NGS	490	26	100	29,253	174,831	16.7	1,051	253	0
	RCA-NGS	768	41	100	241,729	550,786	43.9	4,213	28	2.6
PRV	PCR-NGS	100	64	100	100,905	427,652	23.6	984	192	0
	RCA-NGS	100	81	100	74,457	102,010	72.9	2,544	89	0.02

^a^Amount of DNA used for NGS per run. ^b^Conversion from nanograms to moles was based on the approximate genome size of each virus (i.e., CDV: 15K bases, SARS-CoV-2: 30K bases), or the approximate average genome size in the case of segmented viruses (LCMV 5K bases, SFTSV: 3K bases, IAV: 1.7K bases, PRV: 2.5K bases). ^c^Accuracy of the consensus sequence in comparison with the reference sequence. ^d^Number of mapped reads, unmapped reads and total reads per run analyzed using NanoPipe, and % mapped reads were calculated by dividing the mapped reads by the total reads. ^e^The mean number of read depth per whole genome. ^f^Minimum number of reads per whole genome. ^g^% Coverage of <100 reads was calculated by dividing the number of bases for which the read depth was less than 100 by the total genome number.

On the other hand, the RCA-NGS method tended to accumulate reads for larger segments in segmented genomes, such as SFTSV and IAV (but not PRV) compared to the PCR-NGS method (Table 4; Figures 5, 6, 8). Sequence mismatches mainly resulted from more than four base stretches of identical bases (i.e., homopolymer stretches), which can be visually confirmed and corrected based on the appearance of sharp valleys in the aligned reads on the NanoPipe alignment viewer. Other mismatches that were not due to the homopolymer stretches were further confirmed by Sanger sequencing. The Sanger sequencing results revealed that the consensus sequences determined in this experiment for all mismatches were correct.

**Table 4:** Number and percentage of reads aligned to each segment.

Virus	Segment	Segment size (bases)	PCR-NGS method		RCA-NGS method	
			Mapped reads^a^	% Mapped reads in total mapped reads^b^	Mapped reads	% Mapped reads in total mapped reads
LCMV	L	7,231	19,098	24.6	199,734	75.0
	S	3,375	58,503	75.4	66,423	25.0
SFTSV	L	6,368	3,915	20.7	265,704	93.9
	M	3,378	12,594	66.6	16,982	6.0
	S	1746	2,413	12.8	244	0.1
IAV	1 (PB2)	2,341	4,351	23.8	33,672	51.3
	2 (PB1)	2,341	4,207	23.0	5,471	8.3
	3 (PA)	2,233	3,181	17.4	22,312	34.0
	4 (HA)	1775	1812	9.9	2,139	3.3
	5 (NP)	1,565	1,489	8.1	905	1.4
	6 (NA)	1,413	1,435	7.9	546	0.8
	7 (M)	1,027	974	5.3	483	0.7
	8 (NS)	890	824	4.5	125	0.2
PRV	L1	3,896	11,141	11.0	3,248	4.3
	L2	3,832	11,873	11.8	6,072	8.1
	L3	3,954	11,141	11.0	4,992	6.6
	M1	2,295	11,737	11.6	6,763	9.0
	M2	2,145	12,314	12.2	5,568	7.4
	M3	1984	8,229	8.1	5,884	7.8
	S1	1,602	8,713	8.6	3,137	4.2
	S2	1,322	8,528	8.4	17,607	23.4
	S3	1,192	10,536	10.4	12,667	16.8
	S4	1,184	6,758	6.7	9,278	12.3

^a^Number of mapped reads analyzed using NanoPipe. ^b^% Mapped reads were calculated by dividing the mapped reads by the total reads.

### 3.4. Short cDNA fragment removal before cDNA amplification

**Figure 9:**
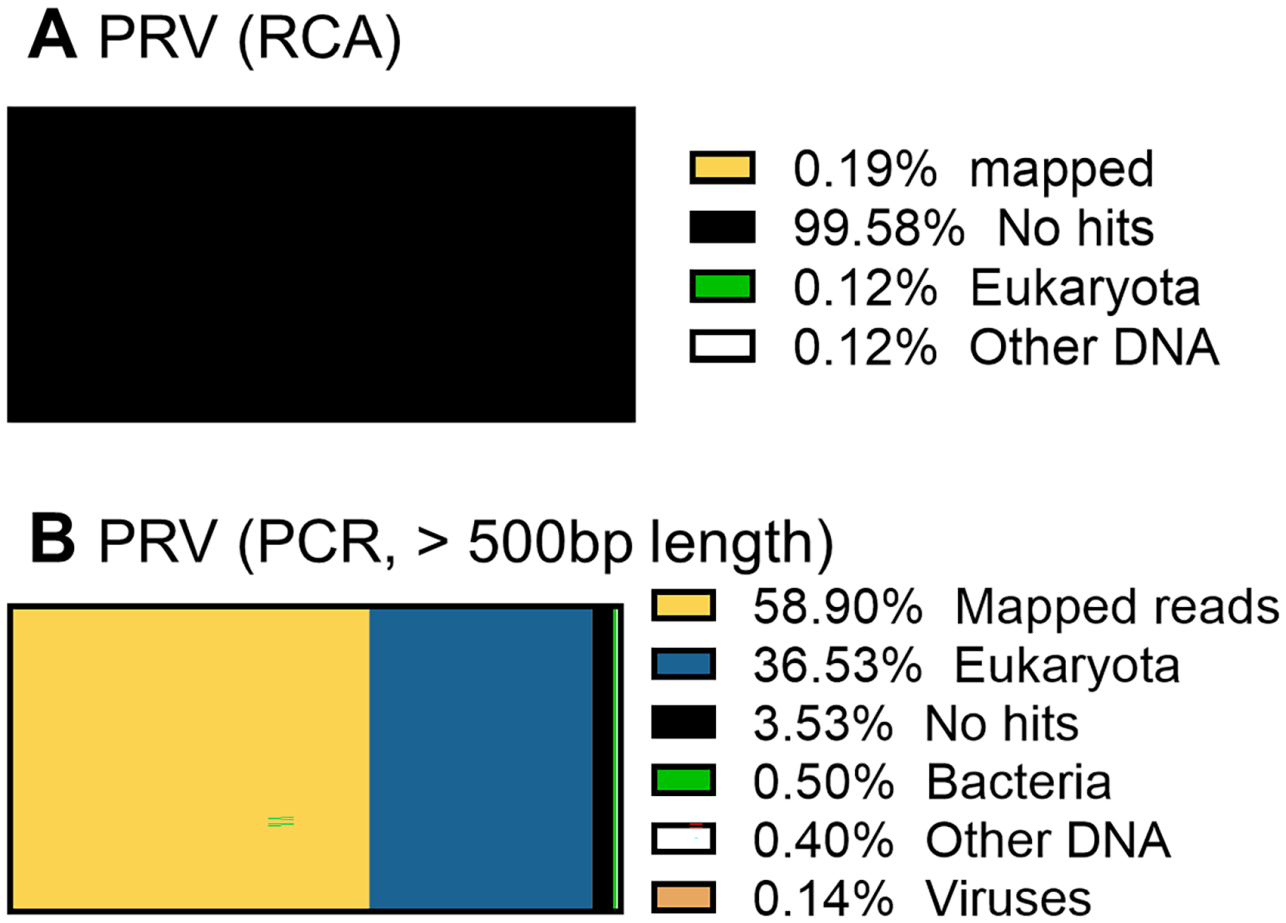
Taxonomic profiling of a PRV working stock without performing short cDNA fragment removal before cDNA amplification. The total reads obtained by the RCA-NGS method (with the exception that short reads were cut off) were analyzed using blastn for NT database (SE) on the Maser data analysis platform and MEGAN **(A)**. The total reads obtained by the PCR-NGS method were analyzed. The total reads obtained by the RCA-NGS method after cutting off the short reads (<500 bp) were also analyzed **(B)**. The percentage of reads categorized to the organism in the total reads is shown as a percentage bar chart, and the actual percentage is shown in the Figure legend.

**Figure 10:**
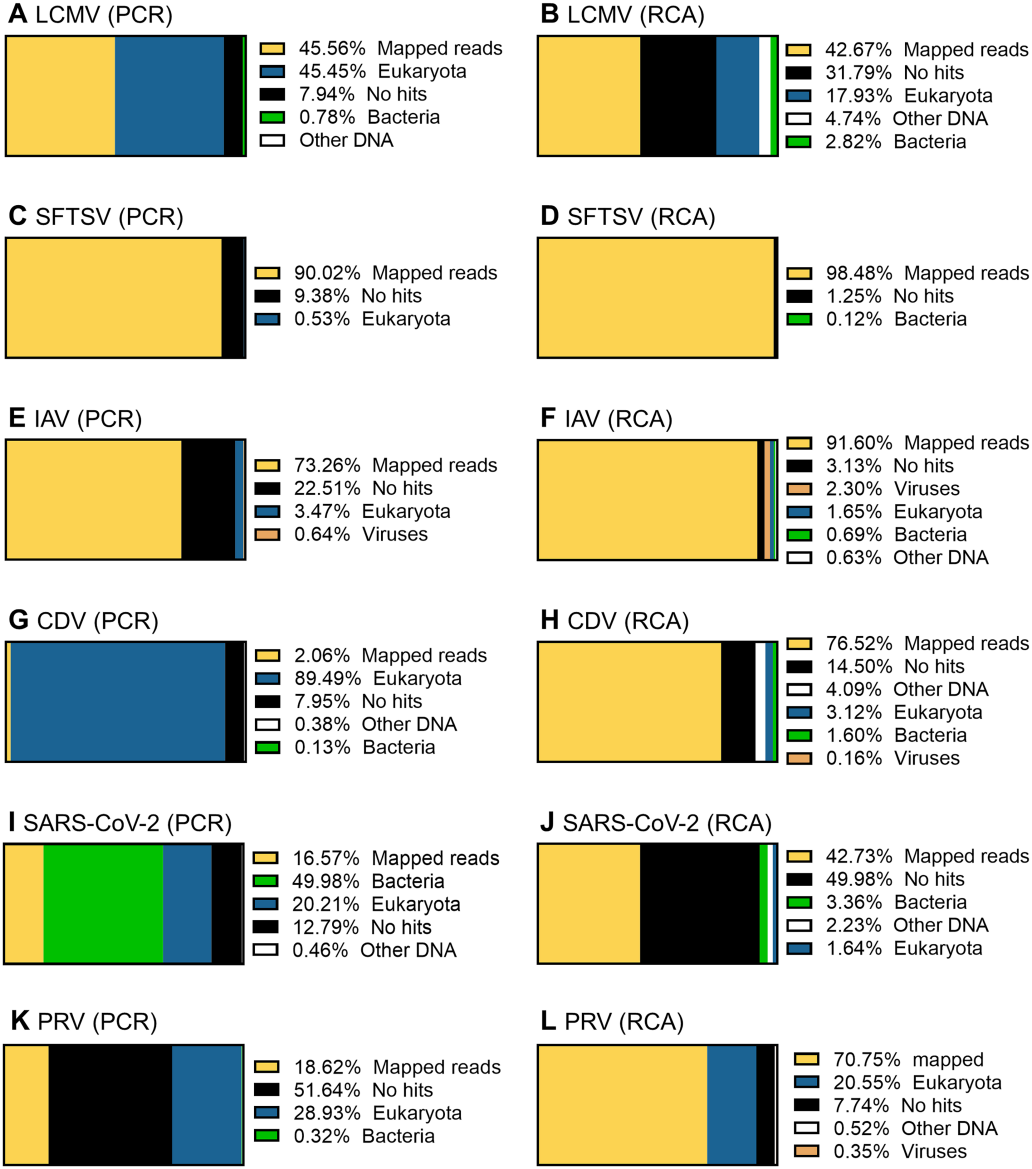
Taxonomic profiling of the working stocks of RNA viruses. The total reads obtained by PCR-NGS method **(A,C,E,G,I,K)** or RCA-NGS method **(B,D,F,H,J,L)** were firstly reference-mapped to LCMV **(A,B)**, SFTSV **(C,D)**, IAV **(E,F)**, CDV **(G,H)**, SARS-CoV-2 **(I,J)** and PRV **(K,L)** using BWA-MEM sequence aligner. Next, unmapped reads were taxonomically profiled using blastn and Megan. The organism that contains more than 0.1% of the total reads is shown in the figure legend.

When the PRV was sequenced using the RCA-NGS method for the first time, only 0.19% (510 reads mapped per 273,796 reads total) of the total reads were mapped in the reference genomes. To investigate the reason for this, the total reads were analyzed using blastn with NCBI-nr database and Megan to clarify the organism that accounted for the majority of the total reads (Figure 9A). Surprisingly, more than 99.5% of the reads matched nothing in the NCBI-nr database. In addition, the percentage of mapped reads drastically improved when the reads obtained by the PCR-NGS method were aligned to the PRV reference genomes with software cutting off short reads (<500 bp) (Figure 9B and Figure 10K). These results suggested that short cDNA fragments may be amplified in the PRV samples and—for some reason—occupied the total reads, which could not be identified in the NCBI-nr database. Hence, a step to remove the short cDNA fragments by adding ×0.8 volume of AMPure XP Reagent with the size-selective property was added to the post-reverse transcription step of the RCA-NGS method. As a result, the mapping rate to the PRV reference genome drastically improved from 0.19 to 72.9%, as shown in Table 3.

### 3.5. Taxonomic profiling

Taxonomic profiling was performed on the successfully sequenced samples to dissect what organisms affected the sequencing. The sequence data shown in Table 3 were reference-mapped again using the BWA-MEM sequence aligner instead of the LAST sequence aligner in NanoPipe since NanoPipe could not extract unmapped reads from the BAM file generated by the NanoPipe analysis. The unmapped FASTQ formatted data were extracted from the reference-mapped BAM files generated by BWA-MEM and were taxonomically profiled using blastn and Megan (Figure 10). The percentage of mapped reads to the reference genome obtained by BWA-MEM was almost the same as the reads mapped by LAST (Table 5). The Eukaryota, which indicates host cell contamination, tended to be more pronounced in the results obtained by sequencing with the PCR-NGS method compared to those obtained by sequencing with the RCA-NGS method in some samples (Figures 10A,B,G–J). The PCR-NGS method revealed bacterial contamination, especially *Mycoplasma* spp. (e.g., *M. hyorhinis*) in the working stock of SARS-CoV-2 (Figure 10I). *M. hyorhinis* contamination was also confirmed using a commercial PCR kit. On the other hand, the contamination was not so pronounced when the RCA-NGS method was used (Figure 10J). Therefore, the PCR-NGS method was more susceptible to mycoplasma contamination than the RCA-NGS method.

**Table 5:** Percentage of mapped reads in total mapped reads.

Virus	NGS method	% Mapped reads^a^	
		Nanopipe (LAST)	BWA-MEM
LCMV	PCR-NGS	46.1	45.6
	RCA-NGS	43.7	42.7
SFTSV	PCR-NGS	90.2	90.0
	RCA-NGS	99.5	98.5
IAV	PCR-NGS	73.8	73.3
	RCA-NGS	92.2	91.6
CDV	PCR-NGS	2.08	2.1
	RCA-NGS	78.5	76.5
SARS-CoV-2	PCR-NGS	16.73	16.6
	RCA-NGS	43.9	42.7
PRV	PCR-NGS	23.6	18.6
	RCA-NGS	72.9	70.7

^a^% Mapped reads were calculated by dividing the mapped reads by the total reads.

## 4. Discussion

In this study, we established novel methods, named PCR-NGS and RCA-NGS, that can determine the whole genome sequence of RNA viruses, including the virus genome termini. These methods can be exploited to determine any whole RNA viral genomes (i.e., single-stranded, double-stranded, positive-stranded, negative-stranded, non-segmented or multi-segmented genomes). In addition, the PCR-NGS and RCA-NGS methods have advantages over conventional methods for determining whole viral genome sequences. First, there is no need for burdensome work, such as the concentration of infectious viruses. Second, even though the concentration step is unnecessary, viral genome-specific primers are not required to amplify the viral cDNA. Third, the terminal sequences of the viral genome are determined without performing RACE. This method should reduce the workload of associated tasks, such as the determination of newly isolated viral genomes, the confirmation of sequences of viruses recovered by reverse genetics, and the management of viruses that have been subjected to a large number of passages.

The result of whole genome sequencing of CDV obtained through the RCA-NGS method was intriguing, given that the working stock was prepared through freeze-thawing in this study. It was expected that the working would contain a higher proportion of host genomes derived from infected cells than other working stocks prepared without freeze-thawing. However, the result showed no discernible impact on host genome contamination, as evidenced by comparison to the results obtained with other viruses (Table 3; Figure 10). These results suggest that the RCA-NGS method is effective for whole-genome sequencing of other RNA viruses that require freeze-thawing in working stock preparation.

However, these methods currently have some limitations. These methods were only confirmed to work with the working stocks that contain isolated RNA viruses at least 5.4 × 10^4^ TCID_50_ per 180 μL. The working stocks generally contain higher virus titers than clinical specimens, while fewer contaminants compete for NGS reads. Therefore, further study will be necessary to apply this technology to clinical specimens to increase the purity of the viral genome by removing the other contaminants in the specimens.

The PCR-NGS and RCA-NGS methods have different advantages in sequencing RNA viruses. In this study, when determining the sequences of RNA viruses containing large genomes, such as CDV and SARS-CoV-2, the percentages of mapped reads obtained by the PCR-NGS method were much lower than those obtained by the RCA-NGS method (Table 3). This difference is probably because the PCR-NGS method requires total amplification from the 5′ end to the 3′ end of the viral genome cDNA. In contrast, when segmented RNA viruses such as SFTSV and IAV were sequenced by the RCA-NGS method, the percentages of mapped reads were much lower than those obtained with the PCR-NGS method, especially in segments of smaller than 2–3 kb in size (Table 4). The RCA-NGS method improved a disadvantage of Phi29 DNA polymerase, which does not efficiently amplify DNA fragments of ≤2 kbp in size (Berthet et al., 2008) by cDNA circularization. However, uncircularized cDNAs of more than 2–3 kb in size were preferably amplified because the circularization efficacy may not be 100%. Therefore, these methods should be used depending on the viral genome organization, and if the organization is unknown, it will be necessary to attempt both methods, although this is laborious.

Oxford Nanopore’s sequencers have difficulty accurately sequencing low-complexity regions, such as homopolymer stretches (Bull et al., 2020). Thus, they exhibit lower accuracy in read-level sequencing than short-read platforms, such as Illumina’s sequencers (Laver et al., 2015; Rang et al., 2018; Bull et al., 2020). Indeed, our sequencing results also sometimes required visual confirmation at the homopolymer portion. Because NanoPipe, which was used in this study for generating consensus sequences, acknowledges the position as uncertain if the nucleotides covered at a position are similar (within 80% similarity) (Shabardina et al., 2019). The methods established in this study overcome the abovementioned instrumental limitation by increasing the read depth by significantly increasing the ratio of targeted viral genomes in the sequencing sample.

In using these methods, particular attention should be paid to mycoplasma contamination in the working stock of viruses. Mycoplasma genomic DNA and the cellular RNA are protected by their own cell membrane and cannot be removed by the nuclease treatment. For instance, the working stock of SARS-CoV-2 was contaminated with mycoplasma used in this study. Therefore, the contamination was reflected in the NGS result, as shown in Figure 10I. Approximately 50% of bacterial reads in the total reads were derived from mycoplasma sequences. Also, when an SFTSV working stock contaminated with mycoplasma was used, the SFTSV-specific reads were only 0.7% (Supplementary Figure S1). Therefore, when the SFTSV working stock was reconstituted in the presence of an anti-mycoplasma antibiotic, the SFTSV-specific reads improved dramatically to 90% (Table 3).

The overall strategy for whole RNA viral genome sequencing using PCR-NGS and RCA-NGS methods applies to isolated viruses, such as clinical isolates, passaged viruses in cells and animals, and viruses rescued by reverse genetics. The methods will solve the time-consuming and complicated process of whole genome sequencing of RNA viruses.

## Data availability statement

The datasets presented in this study can be found in online repositories. The names of the repository/repositories and accession number(s) can be found below: https://www.ddbj.nig.ac.jp/, LC662537, LC662538, LC662539, LC662540, LC662541, LC662542, LC662543, LC662544, PRJDB15039.

## Author contributions

MM: methodology, investigation, formal analysis, data curation, and writing -conceptualization, original draft. TY: supervision, conceptualization, methodology, investigation, formal analysis, data curation, funding acquisition, and writing – original draft. SS, YT, TK, and MSh: writing – review and editing. YO and MY: writing – review and editing, formal analysis, and data curation. HE: supervision, resources, writing – review and editing. MSa: conceptualization, funding acquisition, supervision, resources, and writing – review and editing. All authors contributed to the article and approved the submitted version.

## Funding

This work was supported by Japan Society for the Promotion of Science (JSPS) KAKENHI (grant no. JP20J22501); the Ministry of Health, Labor and Welfare of Japan (grant no. 20HA2005); and Japan Agency for Medical Research and Development (AMED) (grant no. 20fk0108081).

## Conflict of interest

The authors declare that the research was conducted in the absence of any commercial or financial relationships that could be construed as a potential conflict of interest.

## Publisher’s note

All claims expressed in this article are solely those of the authors and do not necessarily represent those of their affiliated organizations, or those of the publisher, the editors and the reviewers. Any product that may be evaluated in this article, or claim that may be made by its manufacturer, is not guaranteed or endorsed by the publisher.
