# Supplementary material for: Rapid whole genome sequencing methods for RNA viruses
Source: Front Microbiol. 2023 Feb 23;14:1137086. doi: 10.3389/fmicb.2023.1137086 (PMC9995502; doi:10.3389/fmicb.2023.1137086)
Supplement: Supplementary file 1 [file Data_Sheet_1.PDF]

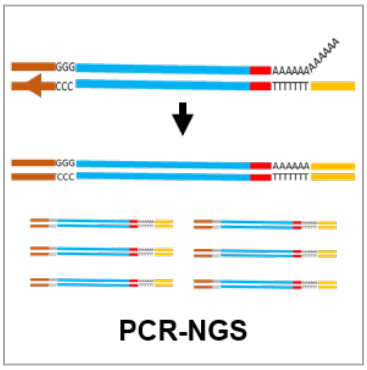

PCR-NGS for RNA viruses

Masayasu Misu[1,2], Tomoki Yoshikawa[1], Satoko Sugimoto[1], Yuki Takamatsu[1], Takeshi Kurosu[1], Yukiteru Ouji[2], Masahide Yoshikawa[2], Masayuki Shimojima[1], Hideki Ebihara[1], Masayuki Saijo[1]

[1]Department of Virology I, National Institute of Infectious Diseases;
[2]Department of Pathogen, Infection and Immunity, Nara Medical University

Masayasu Misu
Department of Virology I, National Institute of Infectious D...

Protocol Info: Masayasu Misu, Tomoki Yoshikawa, Satoko Sugimoto, Yuki Takamatsu, Takeshi Kurosu, Yukiteru Ouji, Masahide Yoshikawa, Masayuki Shimojima, Hideki Ebihara, Masayuki Saijo . PCR-NGS for RNA viruses. **protocols.io**
https://protocols.io/view/pcr-ngs-for-rna-viruses-cnfavbie

Created: Jan 27, 2023

Last Modified: Feb 02, 2023

PROTOCOL integer ID: 75970

Keywords: Oxford Nanopore Technology, RNA virus, Sequence method, MinION, Nanopore sequencing, cDNA-PCRseq, PCR-NGS

ABSTRACT

This PCR-NGS were optimized for an NGS machine, MinION. These methods do not require nucleic acid amplification with virus-specific PCR primers, physical viral particle enrichment, and RACE.

These methods enable whole RNA viral genome sequencing by combining the following techniques:
1) Removal of unwanted DNA and RNA other than the RNA viral genome by nuclease treatment.
2) The terminal of viral genome sequence determination by barcoded linkers ligation.
3) Amplification of the viral genomic cDNA using ligated linker sequences-specific PCR.

This method can be exploited to determine any whole RNA viral genomes (i.e., single-stranded, double-stranded, positive-stranded, negative-stranded, non-segmented or multi-segmented genomes).

MATERIALS

- Micrococcal Nuclease - 320,000 gel units **New England Biolabs Catalog #M0247S**
- High Pure Viral RNA Kit **Roche Catalog #11858882001**
- Turbo DNA-free Kit **Invitrogen - Thermo Fisher Catalog #AM1907**
- **NucleoSpin RNA Clean-up XS - Takara, Catalog #740903.10**
- T4 RNA Ligase 2, truncated KQ - 2,000 units **New England Biolabs Catalog #M0373S**
- **The barcode-polyA linker DNA (e.g., The cSP6-polyA linker DNA)**
- **PCR barcoding kit - Oxford Nanopore Technologies Catalog #SQK-PBK004**
- **cDNA-PCR Sequencing kit - Oxford Nanopore Technologies Catalog #SQK-PCS109**
- Deoxynucleotide (dNTP) Solution Mix **New England Biolabs Catalog #N0447S**
- Superase-In RNase Inhibitor **Thermofisher Catalog #AM2694**
- **Maxima H Minus Reverse Transcriptase - Life Technologies, Catalog #EP0752**
- Agencourt AMPure XP **Beckman Coulter Catalog #A63880**
- **KOD One PCR Master Mix - TOYOBO Catlog #KMM-101**
- Q5 Hot Start High-Fidelity 2X Master Mix - 100 rxns **New England Biolabs Catalog #M0494S**
- Exonuclease I Reaction Buffer - 6.0 ml **New England Biolabs Catalog #B0293S**
- Qubit 4 Fluorometer **Thermo Fisher Scientific Catalog #Q33238**
- Qubit 1X dsDNA HS Assay Kit **Thermo Fisher Scientific Catalog #Q3323[illegible]**
- DNA LoBind Tube 1.5ml **Eppendorf Catalog #022431021**
- 0.2 ml PCR Tube strips **Eppendorf Catalog #0030124359**
- **70 % ethanol**
- **TE(pH8.0)**
- **nuclease-free H_2O**

SAFETY WARNINGS

Follow your facility's regulations and biosafety practices.

BEFORE START INSTRUCTIONS

This method was only confirmed to work with the working stocks that contain isolated RNA viruses at least 3.0×10^5 $TCID_{50}$ per ml.

It is recommended to check no bacterial contamination(e.g., *Mycoplasma* spp.).

Preparation for virus supernatant

1 Centrifuge the working stock virus to remove debris. 10m

6000 x g, Room temperature, 00:10:00

2 Transfer 180 µL virus supernatant to a 1.5ml screw cap tube.

3 Unwanted DNA and RNA mainly originating from the virus-infected cells are digested using Micrococcal Nuclease - 320,000 gel units **New England Biolabs Catalog #M0247S** .

3.1 Total 201 µl reaction 1h

- 180 µL virus supernatant
- 20 µL 10X Micrococcal Nuclease Reaction Buffer
- 1 µL Micrococcal nuclease

Mix by pipetting and spin down.

37 °C water bath 01:00:00

The viral RNA extraction

4 The viral genomic RNA extraction is performed using High Pure Viral RNA Kit **Roche Catalog #11858882001** .

4.1 Add 400 µL of binding buffer (with 4 µL PolyA carrier RNA). 10m

Mix gently by ~5 times pipetting and flicking thoroughly the tube, and spin down.

Room temperature 00:10:00

4.2 Transfer the sample to a High Pure Filter Tube. 1m

8000 x g, Room temperature, 00:01:00

Discard the flow-through liquid and Collection Tube, and insert the Filter Tube into a new Collection Tube.

4.3 Add 500 µL of inhibitor removal bo transfer the sample to a High Pure Filter Tube. 1m

8000 x g, Room temperature, 00:01:00

Discard the flow-through liquid and Collection Tube, and insert the Filter Tube into a new Collection Tube.

4.4 Add 450 µL of wash buffer. 1m

8000 x g, Room temperature, 00:01:00

Discard the flow-through liquid and Collection Tube, and insert the Filter Tube into a new Collection Tube.

4.5 Add 450 µL of wash buffer. 1m

13000 x g, Room temperature, 00:01:00 and discard the flow-through liquid.

Discard the Collection Tube and insert the Filter Tube into a 1.5 ml tube(DNA LoBind Tube 1.5ml **Eppendorf Catalog #022431021**).

4.6 Add 50 µL Elution Buffer. 1m

13000 x g, Room temperature, 00:01:00

Note

The eluted RNA can be stored at -80°C.

Remove unwanted DNA

5 Unwanted DNA mainly from the virus-infected cells in the RNA sample is digested using a Turbo DNA-free Kit **Invitrogen - Thermo Fisher Catalog #AM1907** .

5.1 Total 56 µl reaction 30m

- 50 µL the eluted RNA
- 5 µL 10X reaction buffer
- 1 µL DNase I

Mix gently by pipetting and spin down.

37 °C 00:30:00

6 The viral RNA is purified using **NucleoSpin RNA Clean-up XS - Takara, Catalog #740903.10.**

6.1 Add equal volume 56 µL of Buffer RCU and mix gently.

6.2 Transfer the sample to a NucleoSpin RNA XS Column. 1m

11000 x g, Room temperature, 00:01:00

6.3 Wash the column by 400 µL Buffer RA3. 1m

11000 x g, Room temperature, 00:01:00

Discard the flow-through liquid and Collection Tube, and insert the NucleoSpin RNA XS Column into a new Collection Tube.

6.4 Wash the column by 200 µL Buffer RA3. 2m

11000 x g, Room temperature, 00:02:00

Discard the flow-through liquid and Collection Tube, and insert the NucleoSpin RNA XS Column into a Nuclease-free Collection Tube(1.5 ml).

6.5 Add 10 µL RNase-free H_2O. 1m

11000 x g, Room temperature, 00:01:00

Transfer the sample to a 0.2 ml PCR tube -

0.2 ml PCR Tube strips **Eppendorf Catalog #0030124359** .

cSP6-polyA Linker DNA ligation

7 The viral RNA is ligated with cSP6-polyA Linker DNA using

T4 RNA Ligase 2, truncated KQ - 2,000 units **New England Biolabs Catalog #M0373S** .

- The RNA is ligated to the 3' end with the barcoded(complementary sequence of SP6 (cSP6)) polyA linker DNA. It is able to identify the 3' terminal viral genome sequence. The PolyA sequence is required for reverse transcription for ONT kit (SQK-SQK-PBK004/PCS109).

Note

The cSP6-polyA linker DNA (5'-5rApp-CTATAGTGTCACCTAAATCAAAAAAAAAAAAAAAAAAAAA-3ddC-3'), which is pre-adenylated at the 5' terminal (5rApp), and consists of the complementary sequence of SP6 (CTATAGTGTCACCTAAATC), oligo (dA) 20, and dideoxycytidine (3ddC) at the 3' terminal, was synthesised for 3' linker ligation by Integrated DNA Technologies (Coralville, IA).

7.1 Total 20 µl reaction 15m

- 10 µL Purified RNA
- 1 µL 10 µM the cSP6-polyA linker DNA
- 2 µL 10X T4 RNA Ligase Reaction Buffer
- 6 µL 50% PEG8000 solution
- 1 µL T4 RNA Ligase 2, truncated KQ

Mix gently by pipetting and spin down.
Incubation 25 °C 00:15:00

8 The viral RNA purification by **NucleoSpin RNA Clean-up XS - Takara, Catalog #740903.10.**
go to step #6

Fill the sample to 100 µl with 80 µl TE (pH 8.0) and add 100 µl (equal volume) of Buffer RCU.

Eluted by 10 µl of RNase-free H_2O and transfer the sample to a 0.2 ml PCR tube.

Reverse transcription with strand-switching, SQK-PBK004/ ...

9 The viral RNA is reverse transcribed using **Maxima H Minus Reverse Transcriptase - Life Technologies, Catalog #EP0752, PCR barcoding kit - Oxford Nanopore Technologies Catalog #SQK-PBK004, cDNA-PCR Sequencing kit - Oxford Nanopore Technologies Catalog #SQK-PCS109.**

The following protocol is modified based on the cDNA-PCR Sequencing protocol

(PCSB_9086_v109_revK_14Aug2019) provided by Oxford Nanopore Technologies website.

Note

<cDNA-PCR Sequencing kit (SQK-PCS109)>
RT primer and strand-switching primer

- VN primer (VNP): 5' - 5phos/ ACTTGCCTGTCGCTCTATCTTCTTTTTTTTTTTTTTTTTTTTVN - 3'

Where V = A, C, or G, and N = A, C, G, or T

- Strand-Switching Primer(SSP): 5' - TTTCTGTTGGTGCTGATATTGCT mGmGmG - 3'

9.1 Set up pre-mixture 1 6m

- 9 µL RNA (~ 50ng)
- 1 µL VN primer (VNP)
- 1 µL 10mM dNTP - Deoxynucleotide (dNTP) Solution Mix **New England Biolabs Catalog #N0447S**

Mix gently by flicking the tube, and spin down.
65 °C 00:05:00 and 4 °C on ice 00:01:00

9.2 Set up pre-mixture 2 2m

- 11 µL pre-mixture 1
- 4 µL 5X RT buffer
- 1 µL nuclease-free H_2O
- 1 µL RNase OUT - Superase-In RNase Inhibitor **Thermofisher Catalog #AM2694**
- 2 µL Strand-Switching Primer(SSP)

Mix gently by flicking the tube, and spin down.
42 °C 00:02:00

9.3 Add 1 µL Maxima H Minus Reverse Transcriptase and mix gently by flicking the tube, and spin down. (Total 20 µl reaction). 1h 35m

42 °C 01:30:00
85 °C 00:05:00

PCR with barcoding

33m 20s

10 PCR enzyme;

KOD One PCR Master Mix - TOYOBO Catlog #KMM-101

or

Q5 Hot Start High-Fidelity 2X Master Mix - 100 rxns **New England Biolabs Catalog #M0494S**

10.1 PCR reaction is as follows:

5m 20s

- 5 µL cDNA
- 3 µL LWB (barcoding primer)
- 42 µL nuclease-free water
- 50 µL PCR enzyme (KOD One / Q5)

The reaction mix should be aliquoted in appropriate portions in accordance with the PCR machine used.

<KOD One PCR Master Mix>

**Step**	**Temperature**	**Time**
Heat Activation	98 °C	00:00:15
30 cycles of 3 steps		
Denaturation	98 °C	00:00:10
Annealing	62 °C	00:00:05
Extension	68 °C	35sec or 5 sec/kb
	68 °C	00:02:00

Note

A 35 sec extension is used for viruses with a genome size of less than 7 kb/segment, whereas a 5 sec/kb is employed in other cases.

<Q5 Hot Start High-Fidelity 2X Master Mix>

Step	Temperature	Time
Heat Activation	98 °C	00:00:30
30 cycles of 3 steps		
Denaturation	98 °C	00:00:10
Annealing	72 °C	00:00:10
Extension	72 °C	40 sec/kb
	72 °C	00:02:00

11 Add 1 µL X 2 tube Exonuclease I (E.coli) - 3,000 units **New England Biolabs Catalog #M0293S** . 30m

37 °C 00:15:00
80 °C 00:15:00

12 The PCR product is purified using Agencourt AMPure XP **Beckman Coulter Catalog #A63880** .
Prepare AMpure XP reagent for use; resuspend by vortexing.
Transfer amplified DNA sample to 1.5ml low binding tube.

12.1 Add 80 µL (X 0.8 volume) AMPure XP reagent and mix by pipetting. 5m
Incubate on rotor mixer.
00:05:00 Room temperature

12.2 Spin down and pellet on a magnet. Wait for 00:01:00 and pipette off the supernatant. 1m

12.3 Wash three times by 200 µL 70 % ethanol and remove the ethanol using a pipette and discard.

12.4 Spin down and pipette off any residual ethanol.

12.5 ▪ Resuspend pellet in 12 µL Elution Buffer (EB). 37 °C 00:03:00 and tapping occasionally. Incubate on rotor mixer. 00:07:00

10m

12.6 Spin down and pellet the beads on the magnet until the elute is clear and colourless.

12.7 Remove retain 12 µL elute into a new tube.

13 DNA concentration is measured using a Qubit 4 Fluorometer with Qubit 1X dsDNA HS Assay Kit **Thermo Fisher Scientific Catalog #Q33230** .

2m

- 199 µL 1X working solution
- 1 µL DNA

Mix by vortexing.

Incubate 00:02:00 Room temperature and measure.

Note

The molar quantity of cDNA in the sample can be converted from the concentration through the utilization of the viral genome length or the mean viral genome length if the viral genome is segmented.

Adaptor Ligation

14 ▪ Add 1 µL of Rapid Adaptor (RAP)(SQK-PBK004, SQK-PCS109) to 11 µL library DNA(total approximately 100 fmol).

5m

Mix gently and incubate Room temperature 00:05:00 .

Sequencing by MinION

15 Sequencing according to the manufacturer's instructions.
