# Supplementary material for: Rapid whole genome sequencing methods for RNA viruses
Source: Front Microbiol. 2023 Feb 23;14:1137086. doi: 10.3389/fmicb.2023.1137086 (PMC9995502; doi:10.3389/fmicb.2023.1137086)
Supplement: Supplementary file 2 [file Data_Sheet_2.PDF]

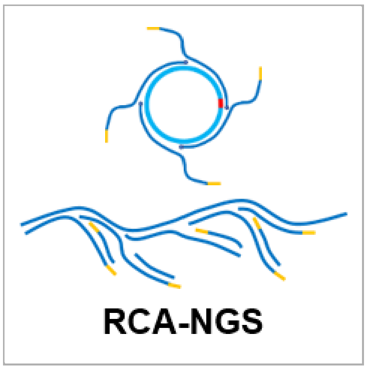

RCA-NGS for RNA viruses

Masayasu Misu[1,2], Tomoki Yoshikawa[1], Satoko Sugimoto[1], Yuki Takamatsu[1], Takeshi Kurosu[1], Yukiteru Ouji[2], Masahide Yoshikawa[2], Masayuki Shimojima[1], Hideki Ebihara[1], Masayuki Saijo[1]

[1]Department of Virology I, National Institute of Infectious Diseases;
[2]Department of Pathogen, Infection and Immunity, Nara Medical University

Masayasu Misu
Department of Virology I, National Institute of Infectious D...

Protocol Info: Masayasu Misu, Tomoki Yoshikawa, Satoko Sugimoto, Yuki Takamatsu, Takeshi Kurosu, Yukiteru Ouji, Masahide Yoshikawa, Masayuki Shimojima, Hideki Ebihara, Masayuki Saijo . RCA-NGS for RNA viruses. **protocols.io** https://protocols.io/view/rca-ngs-for-rna-viruses-cng2vbye

Created: Jan 29, 2023

Last Modified: Feb 02, 2023

PROTOCOL integer ID: 76026

Keywords: Oxford Nanopore Technology, RNA virus, Sequence method, MinION, Nanopore sequencing, RCA-NGS

ABSTRACT

This RCA-NGS were optimized for an NGS machine, MinION. These methods do not require nucleic acid amplification with virus-specific PCR primers, physical viral particle enrichment, and RACE.

These methods enable whole RNA viral genome sequencing by combining the following techniques:
1) removal of unwanted DNA and RNA other than the RNA viral genome by nuclease treatment
2) the terminal of viral genome sequence determination by barcoded linkers ligation
3) Amplification of the viral genomic cDNA using an isothermal DNA amplification technique, such as rolling circle amplification (RCA).

This method can be exploited to determine any whole RNA viral genomes (i.e., single-stranded, double-stranded, positive-stranded, negative-stranded, non-segmented or multi-segmented genomes).

MATERIALS

- Micrococcal Nuclease - 320,000 gel units **New England Biolabs Catalog #M0247S**
- High Pure Viral RNA Kit **Roche Catalog #11858882001**
- Turbo DNA-free Kit **Invitrogen - Thermo Fisher Catalog #AM1907**
- **NucleoSpin RNA Clean-up XS - Takara, Catalog #740903.10**
- T4 RNA Ligase 2, truncated KQ - 2,000 units **New England Biolabs Catalog #M0373S**
- **The barcode-polyA linker DNA (e.g., The cSP6-polyA linker DNA)**
- Superscript IV **Thermo Fisher Scientific Catalog #18090050**
- **SP6 primer (e.g., 5' phosphorylated SP6 primer)**
- Deoxynucleotide (dNTP) Solution Mix **New England Biolabs Catalog #N0447S**

- Superase-In RNase Inhibitor **Thermofisher Catalog #AM2694**
- **Dr.GenTLE Precipitation Carrier - Takara Catalog #9094**
- RNase H - 250 units **New England Biolabs Catalog #M0297S**
- Agencourt AMPure XP **Beckman Coulter Catalog #A63880**
- **CircLigase II ssDNA Ligase - Biosearch Technologies Catalog #CL9021K**
- **GenomiPhi V3 Ready-To-Go DNA Amplification Kit - Cytiva Catalog #25-6601-24**
- T7 Endonuclease I - 250 units **New England Biolabs Catalog #M0302S**
- NEBNext FFPE DNA Repair Mix - 24 rxns **New England Biolabs Catalog #M6630S**
- NEBNext Ultra II End Repair/dA-Tailing Module - 24 rxns **New England Biolabs Catalog #E7546S**
- Blunt/TA Ligase Master Mix - 50 rxns **New England Biolabs Catalog #M0367S**
- NEBNext Quick Ligation Module - 20 rxns **New England Biolabs Catalog #E6056S**
- **Ligation Sequencing Kit - Oxford Nanopore Technologies Catalog #SQK-LSK109**
- **Native Barcoding Expansion - Oxford Nanopore Technologies Catalog #EXP-NBD104, #EXP-NBD114**
- Qubit 4 Fluorometer **Thermo Fisher Scientific Catalog #Q33238**
- Qubit 1X dsDNA HS Assay Kit **Thermo Fisher Scientific Catalog #Q3323[illegible]**
- DNA LoBind Tube 1.5ml **Eppendorf Catalog #022431021**
- 0.2 ml PCR Tube strips **Eppendorf Catalog #0030124359**
- **100 % ethanol**
- **70 % ethanol**
- **TE(pH8.0)**
- **nuclease-free H_2O**

SAFETY WARNINGS

Follow your facility's regulations and biosafety practices.

BEFORE START INSTRUCTIONS

This method was only confirmed to work with the working stocks that contain isolated RNA viruses at least 3.0×10^5 $TCID_{50}$ per ml.

It is recommended to check no bacterial contamination(e.g., *Mycoplasma* spp.).

Preparation for virus supernatant

1 Centrifuge the working stock virus to remove debris. 10m

6000 x g, Room temperature, 00:10:00

2 Transfer 180 µL virus supernatant to a 1.5ml screw cap tube.

3 Unwanted DNA and RNA mainly originating from the virus-infected cells are digested using Micrococcal Nuclease - 320,000 gel units **New England Biolabs Catalog #M0247S** .

3.1 Total 201 µl reaction 1h

- 180 µL virus supernatant
- 20 µL 10X Micrococcal Nuclease Reaction Buffer
- 1 µL Micrococcal nuclease

Mix by pipetting and spin down.

37 °C water bath 01:00:00

The viral genomic RNA extraction

4 The viral genomic RNA extraction is performed using High Pure Viral RNA Kit **Roche Catalog #11858882001** .

4.1 Add 400 µL of binding buffer (with 4 µL PolyA carrier RNA). 10m

Mix gently by ~5 times pipetting and flicking thoroughly the tube, and spin down.

Room temperature 00:10:00

4.2 Transfer the sample to a High Pure Filter Tube. 1m

8000 x g, Room temperature, 00:01:00

Discard the flow-through liquid and Collection Tube, and insert the Filter Tube into a new Collection Tube.

4.3 Add 500 µL of inhibitor removal bo transfer the sample to a High Pure Filter Tube. 1m

8000 x g, Room temperature, 00:01:00

Discard the flow-through liquid and Collection Tube, and insert the Filter Tube into a new Collection Tube.

4.4 Add 450 µL of wash buffer. 1m

8000 x g, Room temperature, 00:01:00

Discard the flow-through liquid and Collection Tube, and insert the Filter Tube into a new Collection Tube.

4.5 Add 450 µL of wash buffer. 1m

13000 x g, Room temperature, 00:01:00 and discard the flow-through liquid.

Discard the Collection Tube and insert the Filter Tube into a 1.5 ml tube - DNA LoBind Tube 1.5ml **Eppendorf Catalog #022431021** .

4.6 Add 50 µL Elution Buffer. 1m

13000 x g, Room temperature, 00:01:00

Note

The eluted RNA can be stored at -80°C.

Remove unwanted DNA

5 Unwanted DNA mainly from the virus-infected cells in the RNA sample is digested using a Turbo DNA-free Kit **Invitrogen - Thermo Fisher Catalog #AM1907** .

5.1 Total 56 µl reaction 30m

- 50 µL the eluted RNA
- 5 µL 10X reaction buffer
- 1 µL DNase I

Mix gently by pipetting and spin down.

37 °C 00:30:00

6 The viral RNA is purified using **NucleoSpin RNA Clean-up XS - Takara, Catalog #740903.10.**

6.1 Add an equal volume 56 µL of Buffer RCU and mix gently.

6.2 Transfer the sample to a NucleoSpin RNA XS Column. 1m

11000 x g, Room temperature, 00:01:00

6.3 Wash the column by 400 µL Buffer RA3. 1m

11000 x g, Room temperature, 00:01:00

Discard the flow-through liquid and Collection Tube, and insert the NucleoSpin RNA XS Column into a new Collection Tube.

6.4 Wash the column by 200 µL Buffer RA3. 2m

11000 x g, Room temperature, 00:02:00

Discard the flow-through liquid and Collection Tube, and insert the NucleoSpin RNA XS Column into a Nuclease-free Collection Tube(1.5 ml).

6.5 Add 10 µL RNase-free H_2O. 1m

11000 x g, Room temperature, 00:01:00

Transfer the sample to a 0.2 ml PCR tube -

0.2 ml PCR Tube strips **Eppendorf Catalog #0030124359** .

cSP6-polyA Linker DNA ligation

7 The viral RNA is ligated with cSP6-polyA Linker DNA using

T4 RNA Ligase 2, truncated KQ - 2,000 units **New England Biolabs Catalog #M0373S** .

The RNA is ligated to the 3' end with the barcoded(complementary sequence of SP6 (cSP6)) polyA linker DNA. It is able to identify the 3' terminal viral genome sequence. The PolyA sequence is required for reverse transcription for ONT kit (SQK-PBK004/ PCS109).

Note

The cSP6-polyA linker DNA (5'-5rApp-CTATAGTGTCACCTAAATCAAAAAAAAAAAAAAAAAAAA-3ddC-3'), which is pre-adenylated at the 5' terminal (5rApp), and consists of the complementary sequence of SP6 (CTATAGTGTCACCTAAATC), oligo (dA) 20, and dideoxycytidine (3ddC) at the 3' terminal, was synthesised for 3' linker ligation by Integrated DNA Technologies (Coralville, IA).

7.1 Total 20 µl reaction 15m

- 10 µL Purified RNA
- 1 µL 10 µM the cSP6-polyA linker DNA
- 2 µL 10X T4 RNA Ligase Reaction Buffer
- 6 µL 50% PEG8000 solution
- 1 µL T4 RNA Ligase 2, truncated KQ

Mix gently by pipetting and spin down.
Incubate 25 °C 00:15:00

8 The linker-ligated viral RNA is purified using **NucleoSpin RNA Clean-up XS - Takara, Catalog #740903.10**

go to step #6

Fill the sample to 100 µl with 80 µl TE (pH 8.0) and add 100 µl (equal volume) of Buffer RCU.

Eluted the RNA in 10 µL RNase-free H_2O and transfer the sample to a 0.2 ml PCR tube.

Reverse transcription

9 The viral RNA is reverse transcribed using Superscript IV **Thermo Fisher Scientific Catalog #18090050** .
5' phosphorylated SP6 primer is used for reverse transcription.

Note

SP6 primer (5' phosphorylated SP6 primer**)**; 5' [Phos]GATTTAGGTGACACTATAG 3'
5' phosphorylation is due to circularization.

9.1 Set up pre-mixture 6m

- 10 µL RNA (~ 50ng)
- 1 µL 50 µM SP6 primer
- 1 µL nuclease-free H_2O
- 1 µL 10mM dNTP - Deoxynucleotide (dNTP) Solution Mix **New England Biolabs Catalog #N0447S**

Mix gently by flicking the tube, and spin down.
65 °C 00:05:00 and 4 °C 00:01:00

9.2 Total 20 µl reaction 20m

- 13 µL pre-mixture sample
- 4 µL 5X SSIV Buffer
- 1 µL 100mM DTT
- 1 µL RNase OUT - Superase-In RNase Inhibitor **Thermofisher Catalog #AM2694**
- 1 µL SuperScript IV Reverse Transcriptase

Mix gently by flicking the tube, and spin down.
55 °C 00:10:00
80 °C 00:10:00

RNase H treatment and ethanol precipitation

20m

10 Add 1 µL RNase H - 250 units **New England Biolabs Catalog #M0297S** .
37 °C 00:20:00 20m

11 Ethanol precipitation using **Dr.GenTLE Precipitation Carrier - Takara Bio Catalog #9094.**

11.1 Add 20 µL TE(pH8.0) to fill up the sample to 40 µL .
.

11.2 Add 4 µL 3M CH_3COONa (pH5.2) .

11.3 Add 1 µL Dr.GenTLE Precipitation Carrier.

11.4 Add 100 µL 100% ethanol.

11.5 13000 x g, Room temperature, 00:15:00 15m

11.6 Discard the supernatant.

11.7 Wash the pellet with 500 µL 70% ethanol. 5m
13000 x g, Room temperature, 00:05:00

11.8 Discard the supernatant and dry.

11.9 Dissolve the pellet in 12 µL nuclease-free H_2O.

(Optional step) Short cDNA fragment removal instead of Et...

12 Short cDNA fragment is removed from the viral RNA sample using

Agencourt AMPure XP **Beckman Coulter Catalog #A63880**

Prepare AMpure XP beads for use; resuspend by vortexing.

Transfer amplified DNA sample to 1.5ml low binding tube.

> **Note**
>
> If a significant proportion of the reads obtained from an NGS run fail to match with the NCBI-nr database (i.e., no hits), it could indicate a large number of short cDNA fragments in the sample. In such instances, re-performing the optional step instead of step 11, such as ethanol precipitation could significantly enhance the outcomes.

12.1 Add 36 µL (X1.8 volume) AMPure XP reagent and mix by pipetting.

Incubate on rotor mixer.

00:05:00 Room temperature

12.2 Spin down and pellet on a magnet.

Wait for 00:01:00 and pipette off the supernatant.

12.3 Wash twice by 200 µL 70 % ethanol and remove the ethanol using a pipette and discard.

12.4 Spin down and pipette off any residual ethanol.

12.5 Resuspend pellet in 20 µL TE(pH 8.0).

37 °C 00:03:00 and tapping occasionally.

Incubate on a rotor mixer.

00:07:00

12.6 Spin down and pellet the beads on the magnet until the elute is clear and colourless.

12.7 Remove retain 20 µL elute into a new tube.

13 Size selection of the cDNA sample is performed using Agencourt AMPure XP **Beckman Coulter Catalog #A63880** .
X0.8 volume of AMPure beads recovers more than 200 bp of nucleic acids.

13.1 Add 16 µL (X0.8 volume) AMPure beads and mix by pipetting.
Incubate on rotor mixer.
00:05:00 Room temperature

13.2 Spin down and pellet on a magnet.
Wait for 00:01:00 and pipette off the supernatant.

13.3 Wash twice by 200 µL 70 % ethanol and remove the ethanol using a pipette and discard.

13.4 Spin down and pipette off any residual ethanol.

13.5 Resuspend pellet in 12 µL nuclease-free water.
37 °C 00:03:00 and tapping occasionally.
Incubate on a rotor mixer.
00:07:00

13.6 Spin down and pellet the beads on the magnet until the elute is clear and colourless.

13.7 Remove retain 12 µL elute into a new tube.

Circularization of cDNA

1h 10m

14 The cDNA is circularized using **CircLigase II ssDNA Ligase - Biosearch Technologies Catalog #CL9021K.**

14.1 Total 20 µl reaction

1h 10m

- 12 µL cDNA
- 2 µL 10X reaction buffer
- 1 µL 50 mM $MnCl_2$
- 4 µL 5M Betaine
- 1 µL CircLigase II

Mix by pipetting and spin down.

60 °C 01:00:00

80 °C 00:10:00

15 Ethanol precipitation using **Dr.GenTLE Precipitation Carrier - Takara Catalog #9094.**

go to step #11

Dissolve the pellet in 10 µL nuclease-free H_2O.

Amplification of cDNA by rolling circle amplification (RCA)

16 cDNA is amplified by Rolling circle amplification (RCA) using **GenomiPhi V3 Ready-To-Go DNA Amplification Kit - Cytiva Catalog #25-6601-24.**

16.1 Total 20 µl reaction

3m

- 10 µL cDNA
- 10 µL 2X denaturation buffer

Mix by pipetting and spin down.

95 °C | 00:03:00

4 °C on ice

16.2 Add 20 µl denatured sample to Ready to go GenomiPhi cake. 4h 10m

30 °C | 04:00:00

65 °C | 00:10:00

17 The cDNA is purified by Agencourt AMPure XP **Beckman Coulter Catalog #A63880**

Prepare AMpure XP beads for use; resuspend by vortexing.

Transfer amplified DNA sample to 1.5ml low binding tube.

17.1 Add 36 µL (X1.8 volume) AMPure beads and mix by pipetting.

Incubate on rotor mixer.

00:05:00 | Room temperature

17.2 Spin down and pellet on a magnet and wait for 00:01:00 and pipette off the supernatant.

17.3 Wash twice by 200 µL 70 % ethanol and remove the ethanol using a pipette and discard.

17.4 Spin down and pipette off any residual ethanol.

17.5 Resuspend pellet in 40 µL nuclease-free H_2O.

37 °C | 00:03:00 and tapping occasionally.

Incubate on a rotor mixer.

00:07:00

17.6 Spin down and pellet the beads on the magnet until the elute is clear and colourless.

17.7 Remove retain 40 µL elute into a new tube.

18 DNA concentration is measured using a Qubit 4 Fluorometer with Qubit 1X dsDNA HS Assay Kit **Thermo Fisher Scientific Catalog #Q33230** .

- 199 µL 1X working solution
- 1 µL DNA

Mix by vortexing.

Incubate 00:02:00 Room temperature and measure.

Note

Confirm the total amplified cDNA to be over 1500 ng, as confirmed using, for instance, a Qubit 4 Fluorometer and Qubit 1X dsDNA HS Assay Kit.

T7 endonuclease treatment

19 The amplified cDNA by RCA is digested using T7 Endonuclease I - 250 units **New England Biolabs Catalog #M0302S** to remove branching.

The following protocol is modified based on the Native barcoding amplicons (with EXP-NBD104, EXPNBD114,and SQK-LSK109) protocol (NBA_9093_v109_revA_12Nov2019) provided by Oxford Nanopore Technologies website.

19.1 Total 30 µl reaction 30m

- x µL (1.0 µg) DNA
- 3 µL NEBuffer 2
- 1.5 µL T7 endonuclease I
- 25-x µL nuclease-free H_2O

Mix by pipetting and spin down.
37 °C 00:30:00

20 The cDNA is purified using Agencourt AMPure XP **Beckman Coulter Catalog #A63880** .

go to step #17 (Add 54 µL (X1.8 volume) AMPure beads)

Resuspend pellet in 24 µL nuclease-free H_2O.

DNA repair and end-prep

21 The purified cDNA is end-prepped using NEBNext FFPE DNA Repair Mix - 24 rxns **New England Biolabs Catalog #M6630S** and NEBNext Ultra II End Repair/dA-Tailing Module - 24 rxns **New England Biolabs Catalog #E7546S**

21.1 Total 30 µl reaction 35m

- 24 µL DNA
- 1.75 µL NEB Next FFPE DNA repair buffer
- 1 µL NEB Next FFPE DNA repair Mix
- 1.75 µL Ultra II end-prep reaction buffer
- 1.5 µL Ultra II end-prep reaction Mix

Mix by pipetting and spin down.

20 °C 00:30:00
65 °C 00:05:00

22 The cDNA is purified using Agencourt AMPure XP **Beckman Coulter Catalog #A63880** .
go to step #17 (Add 54 µL (X1.8 volume) AMPure beads)

Resuspend pellet in 30 µL nuclease-free H_2O.

23 DNA concentration is measured using a Qubit 4 Fluorometer with Qubit 1X dsDNA HS Assay Kit **Thermo Fisher Scientific Catalog #Q33230** . 2m

- 199 µL 1X working solution
- 1 µL DNA

Mix by vortexing.

Incubate 00:02:00 Room temperature and measure.

Note

Confirm the purified cDNA to be approximately 700 ng or more using, for instance, Qubit 4 Fluorometer with a Qubit 1X dsDNA HS Assay Kit.

Native barcode ligation

24 The end-prepped cDNA is ligated with native barcode using **Native Barcoding Expansion - Oxford Nanopore Technologies Catalog #EXP-NBD104, #EXP-NBD114 and** Blunt/TA Ligase Master Mix - 50 rxns **New England Biolabs Catalog #M0367S** .

24.1 Total 25 µl reaction

20m

- x µL DNA(about 400ng)
- 1.5 µL native barcode
- 12.5 µL Blunt/TA ligase master mix
- 11-x µL nuclease-free H_2O

Mix by pipetting and spin down.

25 °C 00:20:00

25 Add 25 µL TE(pH8.0).

26 The cDNA is purified using Agencourt AMPure XP **Beckman Coulter Catalog #A63880** .

26.1 Add 40 µL (X0.8 volume) AMPure XP reagent and mix by pipetting.
Incubate on a rotor mixer.

00:05:00 Room temperature

26.2 Spin down and pellet on a magnet. wait for 00:01:00 .
Pipette off the supernatant.

26.3 Wash twice by 200 µL 70 % ethanol and remove the ethanol using a pipette and discard.

26.4 Spin down and pipette off any residual ethanol.

26.5 Resuspend pellet in 20 µL nuclease-free water.
37 °C 00:03:00 and tapping occasionally.
Incubate on a rotor mixer.
00:07:00

26.6 Spin down and pellet the beads on the magnet until the elute is clear and colourless.

26.7 Remove retain 20 µL elute into a new tube.

27 DNA concentration is measured using a Qubit 4 Fluorometer with
Qubit 1X dsDNA HS Assay Kit **Thermo Fisher Scientific Catalog #Q33230** .

- 199 µL 1X working solution
- 1 µL DNA

Mix by vortexing.

Incubate 00:02:00 Room temperature and measure.
Convert nanogram(ng) into femtomole(fmol) by a calculator.

Note

The molar concentration of the cDNA sample can be converted based on the length of the major band confirmed by electrophoresis after T7 endonuclease treatment. Typically, the fragment lengths are around 2000 bases pairs.

Adaptor ligation

20m

28 Pool each barcoded sample into a 0.2ml PCR tube (Total 100–200 fmol).

29 Adaptor Ligation with pooled samples is performed using **Ligation Sequencing Kit - Oxford Nanopore Technologies Catalog #SQK-LSK109, Native Barcoding Expansion - Oxford Nanopore Technologies Catalog #EXP-NBD104, #EXP-NBD114 and** NEBNext Quick Ligation Module - 20 rxns **New England Biolabs Catalog #E6056S** .

29.1 Total 50 µl reaction

20m

- x µL DNA (100-200 fmol)
- 2.5 µL Adaptor Mix II
- 10 µL NEB Next Quick Ligation Reaction Buffer(5X)
- 5 µL Quick T4 DNA ligase
- 32.5-x µL nuclease-free H_2O

mix gently and incubate.
25 °C 00:20:00

30 The adaptor-ligated cDNA is purified using Agencourt AMPure XP **Beckman Coulter Catalog #A63880** .
Prepare AMpure XP beads for use; resuspend by vortexing.
Transfer amplified DNA sample to 1.5ml low binding tube.

30.1 Add 80 µL AMPure XP reagent and mix by pipetting.
Incubate on a rotor mixer.
00:05:00 Room temperature

30.2 Spin down and pellet on a magnet. Wait for 00:01:00 and pipette off the supernatant.

30.3
- Wash twice by 200 µL . Short Fragment Buffer(SFB) and remove the ethanol using a pipette and discard.

30.4 Spin down and pipette off any residual SFB.

30.5
- Resuspend pellet in 12 µL Elution Buffer (EB)

37 °C 00:03:00 and tapping occasionally.

Incubate on a rotor mixer.

00:07:00

30.6 Spin down and pellet the beads on the magnet until the elute is clear and colourless.

30.7 Remove retain 12 µL elute into a new tube.

31 DNA concentration is measured using a Qubit 4 Fluorometer with

Qubit 1X dsDNA HS Assay Kit **Thermo Fisher Scientific Catalog #Q33230** .

- 199 µL 1X working solution
- 1 µL DNA

Mix by vortexing.

Incubate 00:02:00 Room temperature and measure.

Sequencing by MinION

32 Sequencing according to the manufacturer's instructions.
