# Supplementary material for: Rapid whole genome sequencing methods for RNA viruses
Source: Front Microbiol. 2023 Feb 23;14:1137086. doi: 10.3389/fmicb.2023.1137086 (PMC9995502; doi:10.3389/fmicb.2023.1137086)
Supplement: Supplementary file 3 [file Data_Sheet_3.docx]

Supplementary Material

Rapid whole genome sequencing methods for RNA viruses

Masayasu Misu^1,2^, Tomoki Yoshikawa^1*^, Satoko Sugimoto^1^, Yuki Takamatsu^1^, Takeshi Kurosu^1^, Yukiteru Ouji^2^, Masahide Yoshikawa^2^, Masayuki Shimojima^1^, Hideki Ebihara^1^, Masayuki Saijo^1^

*** Correspondence:** Tomoki Yoshikawa: ytomoki@niid.go.jp

## Supplementary Figure


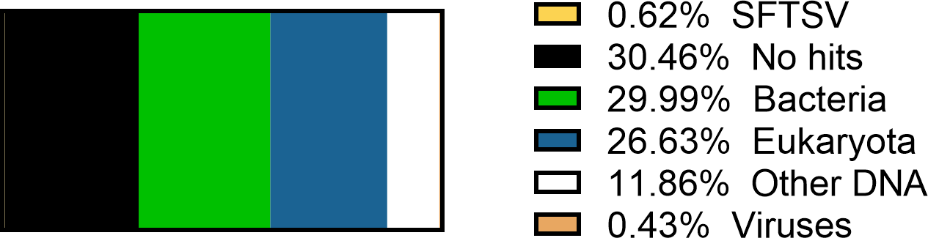


## Supplementary Figure 1. Taxonomic profiling of a working stock of SFTSV. The total reads obtained by the PCR-NGS method were analysed using taxonomic profiling platforms, as described in Fig. 9, 10.
